# Epigenetic silencing of CREB3L1 by DNA methylation is associated with high-grade metastatic breast cancers with poor prognosis and is prevalent in triple negative breast cancers

**DOI:** 10.1186/s13058-016-0672-x

**Published:** 2016-01-25

**Authors:** Alison K. Ward, Paul Mellor, Shari E. Smith, Stephanie Kendall, Natasha A. Just, Frederick S. Vizeacoumar, Sabuj Sarker, Zoe Phillips, Riaz Alvi, Anurag Saxena, Franco J. Vizeacoumar, Svein A. Carlsen, Deborah H. Anderson

**Affiliations:** Cancer Research Group, University of Saskatchewan, 107 Wiggins Road, Saskatoon, SK S7N 5E5 Canada; Epidemiology and Performance Measurement, Saskatchewan Cancer Agency, 4-2105 8th Street, Saskatoon, SK S7H 0T8 Canada; Department of Pathology and Lab Medicine, Royal University Hospital, 2841 – 103 Hospital Drive, Saskatoon, SK S7N 0W8 Canada; Cancer Research, Saskatchewan Cancer Agency, 107 Wiggins Road, Saskatoon, SK S7N 5E5 Canada

**Keywords:** Prognostic marker, Triple negative breast cancer, CREB3L1, DNA methylation, Unfolded protein response

## Abstract

**Background:**

CREB3L1 (cAMP-responsive element-binding protein 3-like protein 1), a member of the unfolded protein response, has recently been identified as a metastasis suppressor in both breast and bladder cancer.

**Methods:**

Quantitative real time PCR (qPCR) and immunoblotting were used to determine the impact of histone deacetylation and DNA methylation inhibitors on CREB3L1 expression in breast cancer cell lines. Breast cancer cell lines and tumor samples were analyzed similarly, and CREB3L1 gene methylation was determined using sodium bisulfite conversion and DNA sequencing. Immunohistochemistry was used to determine nuclear versus cytoplasmic CREB3L1 protein. Large breast cancer database analyses were carried out to examine relationships between CREB3L1 gene methylation and mRNA expression in addition to CREB3L1 mRNA expression and prognosis.

**Results:**

This study demonstrates that the low CREB3L1 expression previously seen in highly metastatic breast cancer cell lines is caused in part by epigenetic silencing. Treatment of several highly metastatic breast cancer cell lines that had low CREB3L1 expression with DNA methyltransferase and histone deacetylase inhibitors induced expression of CREB3L1, both mRNA and protein. In human breast tumors, CREB3L1 mRNA expression was upregulated in low and medium-grade tumors, most frequently of the luminal and HER2 amplified subtypes. In contrast, CREB3L1 expression was repressed in high-grade tumors, and its loss was most frequently associated with triple negative breast cancers (TNBCs). Importantly, bioinformatics analyses of tumor databases support these findings, with methylation of the CREB3L1 gene associated with TNBCs, and strongly negatively correlated with CREB3L1 mRNA expression. Decreased CREB3L1 mRNA expression was associated with increased tumor grade and reduced progression-free survival. An immunohistochemistry analysis revealed that low-grade breast tumors frequently had nuclear CREB3L1 protein, in contrast to the high-grade breast tumors in which CREB3L1 was cytoplasmic, suggesting that differential localization may also regulate CREB3L1 effectiveness in metastasis suppression.

**Conclusions:**

Our data further strengthens the role for CREB3L1 as a metastasis suppressor in breast cancer and demonstrates that epigenetic silencing is a major regulator of the loss of CREB3L1 expression. We also highlight that CREB3L1 expression is frequently altered in many cancer types suggesting that it could have a broader role in cancer progression and metastasis.

**Electronic supplementary material:**

The online version of this article (doi:10.1186/s13058-016-0672-x) contains supplementary material, which is available to authorized users.

## Background

CREB3L1 is a member of the CREB/ATF family of transcription factors and functions as a transducer of the unfolded protein response (UPR) [1]. A large fraction of proteins synthesized in the cell undergo folding and post-translational modification in the endoplasmic reticulum before being released to perform their desired function. This process can be disrupted by endoplasmic reticulum stress resulting from hypoxia, glucose or nutrient depletion, change in calcium homeostasis, or expression of mutant or misfolded proteins, and can lead to the accumulation of unfolded proteins that if released from the endoplasmic reticulum can have detrimental effects. The accumulation of unfolded proteins in the lumen of the endoplasmic reticulum initiates the UPR. The UPR works to regain endoplasmic reticulum homeostasis by reducing protein translocation into the endoplasmic reticulum, increasing the protein-folding capacity of this organelle, decreasing translation initiation, and increasing protein degradation [2]. Prolonged activation of the UPR leads to apoptosis [3].

There are three main transducers of the UPR, namely activating transcription factor-6 (ATF6), inositol requiring 1 (IRE1), and PRK-like endoplasmic reticulum kinase (PERK). Under non-stress conditions they are held in their inactive form by association with chaperone proteins, such as GRP78, bound to their endoplasmic reticulum luminal domain. As unfolded proteins accumulate in the endoplasmic reticulum, GRP78 disassociates from ATF6, IRE1, and PERK, and binds to the hydrophobic regions of unfolded proteins, which are subsequently either refolded, or ubiquitinated and degraded [4]. Activated IRE1 cleaves the mRNA of X-box binding protein 1 (XBP1). The spliced form of XBP1 is translated into a potent transcriptional activator that stimulates the transcription of UPR target genes. PERK phosphorylates eIF2α, which in turn causes a global reduction in mRNA translation. Paradoxically, phosphorylated eIF2α also selectively promotes the translation of specific mRNAs, such as ATF4 [5], which activates the transcription of genes involved in amino acid metabolism and apoptosis [6].

CREB3L1, also termed OASIS (old astrocyte specifically-induced substance) in mice, is the most recently identified member of the UPR containing both a bZIP domain and a DNA binding domain [1]. *CREB3L1* is located on chromosome 11, a chromosome that contains a number of loci that are frequently altered in breast cancer [7–9]. It is an endoplasmic reticulum transmembrane protein and activated in a similar manner to ATF6, via Site-1-protease (S1P) and S2P cleavage in the Golgi apparatus followed by translocation to the nucleus [10]. This mature activated form is a transcription factor, acting on both endoplasmic reticulum stress responsive elements (ESRE) and cyclic AMP responsive elements (CRE) to increase expression of target genes such as *GRP78* [11].

A number of studies have identified roles for the members of the UPR in breast cancer development, progression and resistance to therapy. PERK expression has been shown to be vital for the initiation and progression of breast cancers. Inhibition of PERK expression in animal models results in an increase in reactive oxygen species leading to increased DNA damage and a halting of the cell cycle [12]. ATF4 activation was shown to confer resistance to the chemotherapy agent taxol in hypoxic tumors [13]. A similar finding demonstrated that increased expression of GRP78 is associated with chemoresistance in breast cancer [14, 15]. XBP1 expression has been linked to resistance to anti-estrogen therapies, including tamoxifen, which is especially problematic as XBP1 is rapidly induced by estrogens [16–18]. Recently XBP1 has been shown to be important in driving TNBC oncogenesis through the formation of transcriptional complexes with hypoxia inducing factor 1α (HIF1α) [19].

Although not specific to breast cancer, CREB3L1, like the other members of the UPR, has also been shown to perform important roles in cancer. Epigenetic downregulation of CREB3L1 mRNA expression by DNA methylation is associated with increased tumor grade and aggressive phenotype in bladder cancer [20]. Also, CREB3L1 has been shown to be necessary for the chemotherapeutic drug doxorubicin to block cell proliferation and may function as a biomarker in predicting response to therapy [21, 22]. Doxorubicin increases ceramide production, which in turn stimulates regulated intramembrane proteolysis of CREB3L1 to its mature active form. CREB3L1 then activates expression of target genes, including *p21*, a cell cycle inhibitor [21, 23]. In addition, CREB3L1 may also play a role in limiting the spread of viral expression as CREB3L1 expression blocks proliferation of virally infected Huh7 cells [24].

Our previous work showed that highly metastatic rat and human breast cancer cell lines had reduced expression of CREB3L1 compared to poorly metastatic breast cancer cell lines [25]. We further showed that re-expression of CREB3L1 reduced the in vitro metastatic cell properties, including cell migration, invasion, survival under hypoxic conditions and anchorage-independent growth. In a rat model of breast cancer, CREB3L1-re-expressing cells initially formed large tumors (>0.5 cm^3^), in which 70 % of them regressed to a nearly undetectable size. None of these rats had metastases as compared to a 90 % metastasis rate for the rats with the corresponding CREB3L1-deficient cells [25]. These results suggest that CREB3L1 plays a key role in suppressing tumorigenesis and metastasis.

In this report, we characterize the expression of CREB3L1 in a large panel of breast cancer and non-cancer cell lines and determine whether epigenetic mechanisms regulate CREB3L1 expression in breast cancer. In addition, we characterize CREB3L1 mRNA expression, gene methylation and protein localization in a large number of human tumor samples. Finally, we expanded our analysis of tumor samples from the Cancer Genome Atlas with associated patient data to derive cancer-specific stage association and predict clinical outcome.

## Methods

### Cell culture

A panel containing 40 breast cancer cell lines (and 4 non-tumorigenic breast cell lines) was obtained from the American Type Culture Collection (ATCC, Manassas, Virginia, USA 30-4500 K). Cells were cultured according to ATCC recommendations for fewer than 6 months from the time of resuscitation. All cell lines were authenticated by the supplier (http://www.ATCC.org).

To examine the impact of DNA methylation and/or histone acetylation on CREB3L1 expression, the human breast cancer cell lines BT20, HCC1806 and MDA-MB-468 cells were treated with a DNA methyltransferase inhibitor, 5-aza-2′-deoxycytidine (DAC) (Sigma Aldrich, Oakville, ON, Canada), and/or a histone deacetylase inhibitor, trichostatin A (TSA) (Sigma Aldrich, Oakville, ON, Canada). Cells were grown to 60–70 % confluency and treated with DAC (1 μM) for 96 hours (changing to fresh DAC-containing media every 24 hours), with or without TSA (1 μM), for the last 18 hours as previously reported [26–28]. Cytotoxicity measurements were carried out using a Cytotox Glo Cytotoxicity assay (Promega, Madison, WI, USA G9290) according to their instructions, and no cytotoxicity was observed at 1 μM TSA. Three independent experiments were performed with triplicate samples, with one set used to prepare DNA, one for RNA and the other lysed for western blot analysis of CREB3L1 protein levels, as detailed below. In some instances, cells were treated with the proteasomal inhibitor, MG132 (Sigma Aldrich, Oakville, ON, Canada), at a concentration of 3 μM for the last 18 hours prior to lysis, to prevent the degradation of CREB3L1 protein and better enable visualization on western blots.

### Breast tumor samples

Sections from 216 human primary breast tumors and corresponding de-identified clinical data were obtained from the Manitoba Breast Tumor Bank (Winnipeg, MB, Canada). Four different tumor types were obtained including: infiltrating ductile, infiltrating colloid, infiltrating lobular and infiltrating papillary. For the purposes of assessing the possible correlation between low CREB3L1 expression and more advanced or aggressive tumor type, we have ordered these tumor types from least to most aggressive (colloid, lobular, ductile, papillary) based on several sources [29–33]. Tumor samples were graded by pathologists at the time of diagnosis based on mitotic count, nuclear pleomorphisms and tubule formation, from low (grade 4) to high (grade 9) according to the Nottingham derivation of the Scarff Bloom Richardson system, in which grades 4 to 5 are low, 6 to 7 are medium, and 8 to 9 are high [34]. The estrogen and progesterone receptor status of the tumors was also provided by the Manitoba Breast Tumor Bank. No data were available for the human epidermal growth factor receptor 2 (HER2) status of these samples. Each section consisted of 40–70 % invasive tissue, with the remainder of the tissue being composed of stroma and fat. Samples were accessed and handled according to approved ethics committee guidelines at both the University of Saskatchewan and the Manitoba Tumor Bank (ethics approval number 14-37).

### Western blot analysis

CREB3L1 protein expression in breast cancer cell lines was quantified by western blot analysis as previously described [35]. Briefly, SDS-PAGE was performed using 50 μg total protein, unless otherwise stated, as determined by Lowry (Sigma Aldrich, Oakville, ON, Canada TP0300). Samples were transferred to nitrocellulose and were probed with CREB3L1 (11235-2-AP from Protein Tech, Rosemont, IL, USA; rabbit, 1:500) or ß-actin (C-4 from Santa Cruz Biotechnology, Dallas, TX, USA; mouse, 1:500) primary antibodies, followed by infrared 680 nm or 800 nm dye-tagged secondary antibodies (LI-COR Biosciences, Lincoln, NE, USA; 200 ng/ml). Blots were imaged with the Odyssey Infrared Imaging System (LI-COR Biosciences, Lincoln, NE, USA), quantified, normalized to a ß-actin loading control, and reported relative to that in MDA-kB2 cells.

### Methylation sequencing

DNA was extracted from breast tumor sections and cell cultures using the QIAamp DNA Mini Kit (Qiagen, Toronto, ON, Canada). Sodium bisulfite treatment was performed using the EpiTect Bisulfite kit (Qiagen, Toronto, ON, Canada) to convert unmethylated cytosine residues to uracil according to the supplier’s instructions. CpGenome Human Methylated DNA Standard, (Cedarlane, Burlington, ON, Canada) and Epitect Control unmethylated DNA (Qiagen, Toronto, ON, Canada) were used as positive and negative controls, respectively.

Primers were designed using Primer3 software [36] to amplify (from bisulfite-treated DNA) two overlapping fragments of *CREB3L1* spanning base pair −492 to +290 relative to the transcription start site (Additional file 1: Table S1). Our preliminary data indicated that DNA methylation was concentrated within the beginning of the coding region, thus, a set of primers that amplify a fragment from −51 to +258 to target this methylation-rich region was used. PCR was performed with 100 ng bisulfite-treated DNA in a 50-μl reaction with the TaKaRa EpiTaq HS kit (Cedarlane, Burlington, ON, Canada) according to the supplier’s instructions. The thermocycling protocol consisted of 45 cycles of 10 seconds at 98 °C, 30 seconds at annealing temperature (Additional file 1: Table S1), and 1 minute at 72 °C. QIAquick PCR Purification Kit (Qiagen, Toronto, ON, Canada) was used to purify PCR products prior to sequencing. Sanger sequencing was performed by the Plant Biotechnology Institute (Saskatoon, SK, Canada) and results were visualized with MacVector version 12.5 software (MacVector, Inc., Apex, NC, USA). Only sequences containing efficient C to T conversions, indicative of effective sodium bisulfite conversion at the non-CpG sites (that would not be methylated) were used for methylation analyses. Sequences were assessed for the presence of methylated cytosine residues at CpG dinucleotide motifs by using a qualitative assessment at each possible methylated position. Methylation was scored as low, but present and given a value of 1, if the C peak was above background noise even if some T was also present. Methylation was scored as high and given a value of 2, if the C peak was the tallest peak observed at that position. Initially sequences were analyzed from −429 to +259, relative to the translational start site, a region that includes 60 CpG sites. As most methylations were concentrated between −15 and +259, subsequent analyses focused on these 24 CpG sites.

### Quantitative real-time PCR

Total RNA was extracted from cell lines using the RNeasy kit (Qiagen, Toronto, ON, Canada) and from breast tumor sections using the PicoPure RNA Isolation kit (Life Technologies). RNA was reverse-transcribed to cDNA using Superscript II Reverse Transcriptase and oligo-dT primers (Invitrogen) according to the supplier’s instructions. CREB3L1 expression was measured by quantitative real-time PCR performed using TaqMan probes (assay ID Hs00999642_m1, Life Technologies, Waltham, MA, USA) and TaqMan Gene Expression Master Mix (Life Technologies, Waltham, MA, USA) according to manufacturer’s protocols. The sequence of these primers is proprietary, but it amplifies a 103-bp fragment at the junction of exons 8 and 9 such that it will only detect the full-length transcript. Relative expression was calculated using expression of glyceraldehyde-3-phosphate dehydrogenase (GAPDH) (assay ID Hs99999905_m1, Life Technologies, Waltham, MA, USA) as a reference gene. Samples were analyzed in triplicate per reaction using the StepOnePlus Real-Time PCR System (Applied Biosystems, Waltham, MA, USA). Results are the mean of two independent reactions and reported as the relative change in expression compared to the MDA-kB2 cell line (for the cell line analysis), or control normal breast tissue sample (Manitoba Breast Tumor Bank, Winnipeg, MB, Canada) for the tumor sample analysis. Where samples were available, the HER2 status of the tumor samples was determined by qPCR as above, but using HER2-specific TaqMan probes (assay ID Hs1001580_m1, Life Technologies, Waltham, MA, USA), relative to a GAPDH reference gene. HER2 status was reported as compared to the MDA-kB2 cell line (HER2-positive, but not amplified) as follows (negative <1, positive = 1–9, amplified >9).

### Copy number variation

CREB3L1 gene copy number was assessed using a digital droplet PCR assay [37]. The number of target fragments in the original sample is calculated using a Poisson distribution and the copy number of the gene of interest is calculated by normalizing it to a reference gene (*AP3B1*) to adjust for global ploidy changes [38].

DNA extracted from human breast tumors was digested with HaeIII (New England Biolabs, Whitby, ON, Canada) prior to analysis, as per supplier’s instructions. To measure *CREB3L1* gene copy number, custom-designed CREB3L1 primers and FAM-labeled TaqMan probe (forward 5′- GATCCAGCTTCCTGGACTTG-3′, reverse 5′-GTAAGATGAAGGGTCTCCGTTC-3′, probe 5′-ACGAGTCGGACTTCCTCAACAATGC-3′; Bio-Rad, Mississaugo, ON, Canada) were combined in a duplex reaction with pre-validated AP3B1 primers and HEX-labeled TaqMan probe (assay ID dHsaCP1000483, Bio-Rad, Mississaugo, ON, Canada) and ddPCR Supermix for Probes (Bio-Rad, Mississaugo, ON, Canada). Droplets were generated using the QX100 Droplet Generator (Bio-Rad, Mississaugo, ON, Canada) and thermocycled to the completion of the following protocol: 10 minutes at 95 °C; 40 cycles of 30 seconds at 94 °C and 1 minute at 60 °C; 10 minutes at 98 °C. Droplets were analyzed using the QX100 Droplet Reader (Bio-Rad, Mississaugo, ON, Canada) and copy numbers were computed with QuantaSoft software (Bio-Rad, Mississaugo, ON, Canada) after normalization to *AP3B1*. Results are reported as the average copy number of *CREB3L1* genes per cell.

### Immunohistochemistry

Immunohistochemical staining of CREB3L1 protein was performed on formalin-fixed paraffin-embedded human breast tumor samples obtained from the Manitoba Tumor Bank, according to the manufacturer’s instructions using Pierce Peroxidase Detection Kit (Thermo Scientific, Burlington, ON, Canada). Briefly, sections were de-paraffinized and heated in citrate buffer (pH = 6), followed by an overnight incubation at 4 °C with a rabbit polyclonal anti-CREB3L1 antibody (1:100; Protein Tech, Rosemont, IL, USA 11235-2-AP). Subsequently, slides were incubated with a horseradish peroxidase goat anti-rabbit secondary antibody (1:1000; Abcam, Toronto, ON, Canada ab6721) for 2 hours at room temperature and reacted with 3,3′-Diaminobenzidine (DAB) for 15 minutes. Sections were counterstained with hematoxylin. CREB3L1 staining was evaluated by a pathologist using two criteria, staining intensity (absent = 0, weak = 1, moderate = 2, strong = 3, very strong = 4) and % cells staining positive (0–5 % = 0, 6–49 % = 1, 50–69 % = 2, 70–89 % = 3, 90–100 % = 4). Scores were added together and described as little or no CREB3L1 staining (combined score 0–1), low (scores 2–3), medium (scores 4–5) and high (scores 6–8) CREB3L1 expression, analogous to the Allred system [39]. In addition, the subcellular location of CREB3L1 was evaluated as nuclear or cytoplasmic.

### *In silico* analysis

Publically available RNA-Seq Version 2 containing normalized gene expression datasets for 24 different cancer types were downloaded from the online database, The Cancer Genome Atlas (TCGA; http://tcga-data.nci.nih.gov). These data contained expression profile, clinical information, tumor stage, and immunohistochemical (IHC) results for the determination of breast cancer subtype for each breast tissue sample. The RNA-seq by expectation-maximization (RSEM) algorithm-normalized gene expression profile was downloaded from TCGA. Although the microarray-based dataset is also available in this database, to exclude problems that could be caused by combining different platforms, measurement types, and normalization procedures, we used only the RNAseqV2 dataset for all our gene expression analyses. To identify different subtypes of breast cancer, we used the IHC annotations available within our downloaded TCGA dataset. This approach was further verified by an additional independent analysis, where the TNBC population was defined as the group of samples with the lowest 10 % of estrogen receptor (ER), progesterone receptor (PR) and HER2 expression. Analysis of these populations confirmed that the same patients matched the IHC category. Similarly, for stage-specific classification, the annotation of each patient from the downloaded TCGA dataset was used. The subtype classifications or stage-specific classifications were analyzed using python scripts with pylab and the scipy stat built-in libraries to generate the graphs showing relationships with methylation and expression. The non-parametric Mann–Whitney *U* test was used to compare two groups. The methylation data were also downloaded from TCGA (Human Methylation 450 K data). Methylation probes were deconvolved for every single region across the promoter, and the intronic and exonic regions. Normalized values were downloaded onto Gene-E to generate the heatmap. Methylation data of either selected regions as identified in Li et al. [40] or as pre-selected by cBioPortal were used to generate correlation analyses between methylation and gene expression.

The relationship between CREB3L1 methylation and expression (Fig. 6c) was analyzed using cBioPortal (http://www.cbioportal.org/index.do) [41, 42]. The dataset analyzed was the breast invasive carcinoma (TCGA Provisional) containing 737 cases, and was accessed on 12 February 2015. Kaplan–Meier survival analysis was carried out using KM-plotter (http://kmplot.com/analysis/) [43]. Gene expression data and relapse-free survival information were downloaded from Gene Expression Omnibus (GEO) (Affymetrix microarrays only), European Genome-phenome Archive (EGA) and TCGA. The database is handled by a PostgreSQL server, which integrates gene expression and clinical data simultaneously. To analyze the prognostic value of a particular gene, the patient samples were split into two groups at the median of the proposed biomarker. The two patient cohorts were compared using a Kaplan–Meier survival plot, and the hazard ratio with 95 % confidence intervals and log-rank *p* value were calculated. The database was accessed on 22 February 2015.

### Statistical analyses

Spearman correlations were determined using free online software [44]. Statistical analyses were performed using SAS version 9.3 (SAS Institute Inc., Cary, NC, USA) software. Significance was set at *p* <0.05 and error reported as plus or minus the standard deviation (SD). The non-parametric Mann–Whitney *U* test was used to compare two groups. The Kruskal–Wallis test was used to compare four or more groups of sample data, using SPSS Statistics 23. Provided that significant differences were detected by the Kruskal–Wallis test, a post-hoc test was performed using pairwise comparisons. Survival analysis was performed using the Kaplan–Meier estimator with the non-parametric log-rank test to measure the equity of strata.

## Results

### CREB3L1 mRNA levels are low in most TNBC cell lines and inversely correlate with *CREB3L1* gene methylation in human breast cancer cell lines

**Fig. 1:**
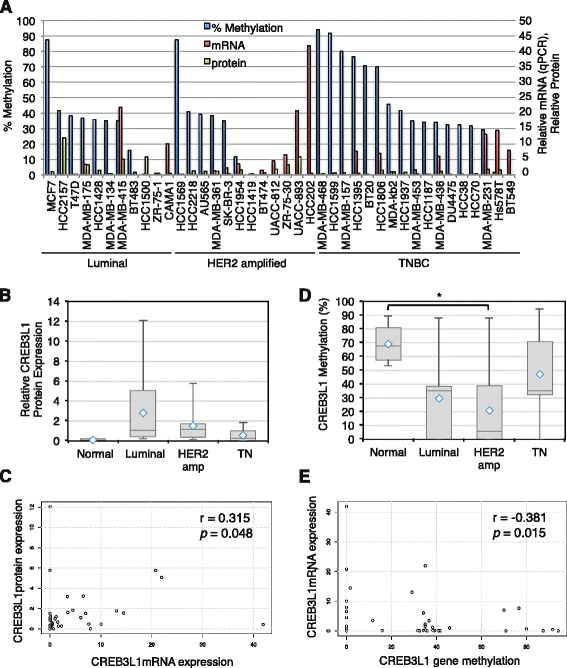
CREB3L1 gene methylation in breast cancer cell lines, and its inverse correlation with CREB3L1 expression. **a** A panel of 40 breast cancer cells were analyzed in parallel for *CREB3L1* gene methylation (% methylation, *blue bars*), mRNA (quantitative PCR (*qPCR*), *red bars*) and protein expression (quantitative western blot, *green bars*). Percent methylation (data from Additional file 5: Table S2; *left axis labels*) were plotted together with the relative CREB3L1 mRNA and protein expression (*right axis labels*). **b** The fraction of cell lines with low CREB3L1 protein expression in each breast cancer subtype. **c** Positive correlation of CREB3L1 protein expression and its mRNA levels in breast cancer cell lines. Spearman correlation (*r* value) and significance *p* value. **d** The fraction of cell lines containing *CREB3L1* DNA methylation in each breast cancer subtype. Statistical differences were analyzed by post-hoc pairwise comparison: *p <0.05. **e** Negative correlation of CREB3L1 mRNA expression and its DNA methylation status in breast cancer cell lines. Spearman correlation (*r* value) and significance *p* value. *HER2* human epidermal growth factor receptor 2, *TNBC* triple negative breast cancer

We analyzed CREB3L1 mRNA expression in a large panel of 40 breast cancer cell lines using quantitative real-time PCR (qPCR; Fig. 1a). In parallel, we measured CREB3L1 protein levels using a quantitative western blot analysis (Additional file 2: Figure S1 and Fig. 1a). CREB3L1 protein was low in four non-tumorigenic normal breast cell lines (184B5, MCF10A, MCF10F and MCF12A), and also in TNBC cell lines, as compared to luminal and HER2 amplified cell lines (Fig. 1b). A positive correlation between CREB3L1 protein and mRNA expression was observed (Spearman coefficient 0.315; *p* = 0.048) (Fig. 1c).

We analyzed the methylation status of the CREB3L1 promoter region, as one possible mechanism that could regulate CREB3L1 expression in breast cancer cells. A large CpG island (672 nucleotides) was identified in the promoter region of the *CREB3L1 *gene, using the University of California, Santa Cruz (UCSC) genome browser [45] (Additional file 3: Figure S2 and Additional file 4: Figure S3a). This CpG island contains 51 CpG sites and extends from −119 nucleotides upstream of the translational start site to 554 nucleotides into the coding region of the gene. In addition, there are 27 CpG sites between the transcriptional start site at −451 and the start of the CpG island (Additional file 3: Figure S2). We decided to analyze a fairly large region using sodium bisulphite-treated DNA and Sanger sequencing [46–48]. This provided semiquantitative methylation data and importantly allowed us to assess a relatively large region including 60 CpG sites between −429 and +259 (Additional file 4: Figure S3b, c). We noted a strong preferential methylation at the 3′ end of this region at CpG sites 238 and 259. Samples with more extensive methylation had additional methylations extending towards the 5′ end of this region (Additional file 5: Table S2).

We found that more than half the breast cancer cell lines contained *CREB3L1* methylated CpG sites; in particular, those of the TNBC subtype of breast cancer were highly methylated (Fig. 1a and d). In addition, four non-tumorigenic normal breast cell lines (184B5, MCF10A, MCF10F and MCF12A) all had many methylated sites in the region analyzed (Additional file 5: Table S2 and Fig. 1d). There was an inverse correlation between *CREB3L1* DNA methylation within this region and CREB3L1 mRNA expression (Fig. 1e; Spearman correlation −0.381, *p* = 0.015). These results suggest that methylation of the CpG sites in the *CREB3L1* gene may in some cases negatively regulate CREB3L1 mRNA expression, particularly in TNBC cell lines.

**Fig. 2:**
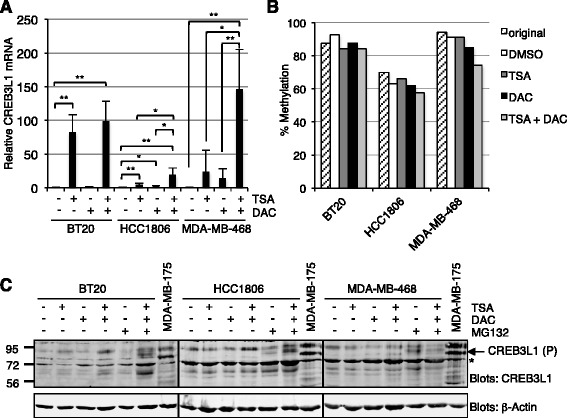
Trichostatin A (*TSA*) and to a lesser extent, 5-aza-2′-deoxycytidine (*DAC*) treatment induces CREB3L1 mRNA expression. **a** BT20, HCC1806 and MDA-MB-468 cells were treated with DAC ± TSA. CREB3L1 mRNA levels were quantified (qPCR) relative to a glyceraldehyde-3-phosphate dehydrogenase (GAPDH)-specific control. Mean ± SD from three independent experiments: *p <0.05; **p <0.01. **b** *CREB3L1* DNA methylation was determined for each sample as for Fig. 1a and plotted with values for the original untreated cell line for comparison. **c** Parallel samples were treated as in **a** and cell lysates (50 μg) were immunoblotted for CREB3L1. Additional samples were also treated where indicated with the proteasomal inhibitor MG132 to block CREB3L1 protein degradation. Lysates from MDA-MB-175 cells were included as a positive control for a cell line expressing endogenous CREB3L1. The full-length precursor form (approximately 84 kDa) of CREB3L1 is indicated with *arrows*; *background band. β-actin levels are shown as loading controls. *DMSO* dimethyl sulfoxide

To investigate the role of epigenetic mechanisms in the regulation of CREB3L1 expression, the impact of the DNA methyltransferase inhibitor, 5-aza-2′-deoxycytidine (DAC), and the histone deacetylase inhibitor, TSA, were tested [49–51]. BT20, HCC1806 and MDA-MB-468 human breast cancer cells were treated with DAC and/or TSA and CREB3L1 mRNA levels were analyzed by qPCR, relative to a GAPDH-specific control (Fig. 2a). TSA strongly induced CREB3L1 mRNA levels in BT20 cells, whereas both TSA and DAC induced CREB3L1 mRNA in HCC1806 and MDA-MB-468 cells. Methylation analysis of *CREB3L1* DNA in these samples showed little or no change in BT20 cells, a small reduction in the HCC1806 cells, and a larger reduction in the MDA-MB-468 cells in response to DAC treatment (Fig. 2b). These results were consistent with the effects of DAC (±TSA) on CREB3L1 mRNA levels, suggesting that in BT20 cells, CREB3L1 is not regulated significantly by DNA methylation, but strongly by histone acetylation. In contrast, CREB3L1 mRNA expression in MDA-MB-468, and to a lesser extent HCC1806 cells, are regulated by both DNA methylation and histone acetylation.

Parallel samples were assessed for CREB3L1 protein levels by CREB3L1 immunoblotting (Fig. 2c) and showed only modest increases upon TSA and/or DAC treatment. As CREB3L1 protein levels have been reported to be downregulated via constitutive ubiquitination and proteasomal degradation in mouse embryo fibroblasts and C6 glioma cells [52], the TSA and/or DAC treatments were repeated including the proteasomal inhibitor, MG132. The presence of MG132 increased CREB3L1 protein levels more robustly (Fig. 2c). These results suggest that both epigenetic and post-translational mechanisms can contribute to reduced CREB3L1 in human breast cancer cells.

### Nuclear CREB3L1 protein is decreased in high-grade breast tumors

**Fig. 3:**
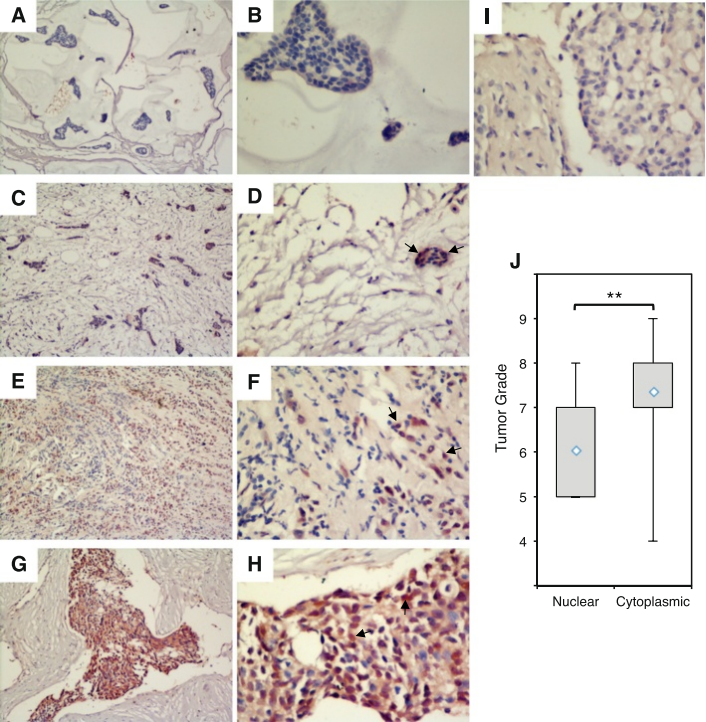
Immunohistochemical analyses of CREB3L1 protein expression in human breast tissue sections. Images were taken using × 10 (**a**, **c**, **e**, **g**) and × 40 (**b**, **d**, **f**, **h**, **i**) objective. Little or no (**a**, **b**), low (**c**, **d**), medium (**e**, **f**) and high (**g**, **h**) CREB3L1-expressing breast tumor samples. **i** A negative control sample where the primary antibody was omitted. **j** Loss of CREB3L1 protein within nuclei was associated with high-grade breast cancers (n = 19 for nuclear; n = 22 for cytoplasmic). *Diamonds* medians, *boxes* 25–75 % quartiles, *vertical lines* range, peak and minimum; **p <0.01

We further extended these studies from human breast cancer cell lines to human breast cancer tissues. Tumor samples included the associated data for ER and PR status, but the HER2 status was unknown. Tumor grade was provided for each sample and was determined using the Nottingham derivation of the Scarff Bloom Richardson system, where grades 4 to 5 are low, 6 to 7 are medium, and 8 to 9 are high [34]. An IHC analysis was carried out to characterize CREB3L1 protein expression and subcellular localization in 97 human breast tumor sections (Fig. 3). Of the 97 samples stained, 56 (58 %) showed little or no CREB3L1 staining (Fig. 3a–b). The samples with CREB3L1 protein staining were further divided into low (n = 9; Fig. 3c–d), medium (n = 14; Fig. 3e–f) and high (n = 18; Fig. 3g–h) based on the intensity and frequency of CREB3L1 protein expression.

As a stress-activated protein, CREB3L1 resides within the cytoplasm as an endoplasmic reticulum transmembrane protein, which can be processed after trafficking to the Golgi to release an active transcription factor that translocates into the nucleus. As has been noted in bladder cancer tumors [20], the 41 breast tumor samples that expressed CREB3L1 protein showed two main CREB3L1 localization patterns. Approximately half (n = 19) had predominantly nuclear CREB3L1 protein localization (Fig. 3c–f), whereas the other half (n = 22) had intense, mainly cytoplasmic staining (Fig. 3g–h). Importantly, the low-grade breast tumors more frequently had nuclear CREB3L1 protein, in contrast to the high-grade breast tumors in which CREB3L1 was cytoplasmic (*p* = 0.003, Mann–Whitney *U* test) (Fig. 3j). These results suggest that when CREB3L1 protein is expressed in breast tumors, the high-grade tumors have lost the nuclear localization of this transcription factor.

To assess whether there was any relationship between different tumor subtypes and either overall CREB3L1 protein expression and/or subcellular localization, we needed to determine their HER2 status. Where sufficient tumor samples were available, we determined the HER2 status using qPCR as detailed in “Methods”. This allowed us to group the tumor samples into molecular subtypes defined as: luminal (ER+ and/or PR+, ± HER2 but not amplified levels), HER2 (HER2 amplified), and TNBC (ER–, PR–, HER2–). In the absence of Ki67 data the luminal subtype was not further stratified into luminal A and luminal B. The amount of some samples was too small to determine the HER2 status, and these were grouped as unknown. There were no TNBC samples in our human tumor specimens, although several of the unknown samples were negative for ER and PR, but unknown for HER2 status.

**Fig. 4:**
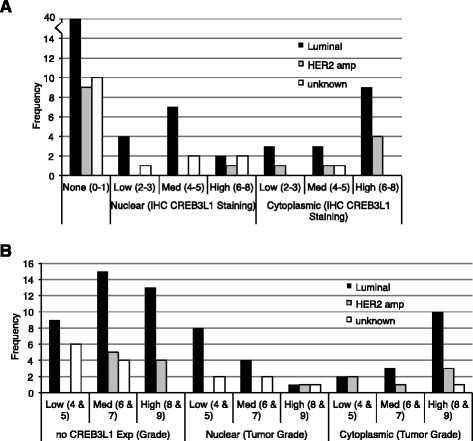
Low-medium (*med*)-grade tumors express low amounts of nuclear CREB3L1 whereas high-grade tumors have high cytoplasmic localization. Breast tumor samples (97) were analyzed by immunohistochemical analysis (IHC) (shown on Fig. 3) and were classified according to IHC staining (**a**) and tumor grade (**b**), subdivided by tumor subtype (luminal, human epidermal growth factor receptor 2 (*HER2*) amplified, unknown) and nuclear versus cytoplasmic localization

For each subtype there were similar numbers of samples with and without CREB3L1 staining (Additional file 6: Figure S4a). Although the sample size within each group was small, there was a larger proportion of HER2 amplified tumor samples with cytoplasmic CREB3L1 protein, rather than nuclear CREB3L1 (Additional file 6: Figure S4b). We also compared the distribution of breast cancer subtypes by their nuclear and cytoplasmic localization and levels of CREB3L1 protein expression (Fig. 4a). Luminal breast cancers were more frequently observed to have low-medium levels of CREB3L1, effectively localized to the nucleus, or high levels of CREB3L1 within the cytoplasm. In contrast, HER2 amplified breast cancers contained mainly high levels of cytoplasmic CREB3L1 (Fig. 4a). In parallel, luminal breast cancers with nuclear CREB3L1 were most frequently of low-medium grade, whereas those with cytoplasmic CREB3L1 were often high-grade tumors (Fig. 4b). This was not observed for the HER2 amplified breast tumors. These results suggest that in HER2 amplified breast cancers about half express CREB3L1, and the CREB3L1 is primarily localized in the cytoplasm where its transcriptional regulation would not be active. Further, in luminal breast cancers, again about half express CREB3L1, that in lower grade tumors is typically nuclear, where it could regulate transcriptional targets, and in higher grade tumors is typically cytoplasmic, where it could not.

### Analysis of CREB3L1 mRNA expression and methylation in human breast tumor samples

**Fig. 5:**
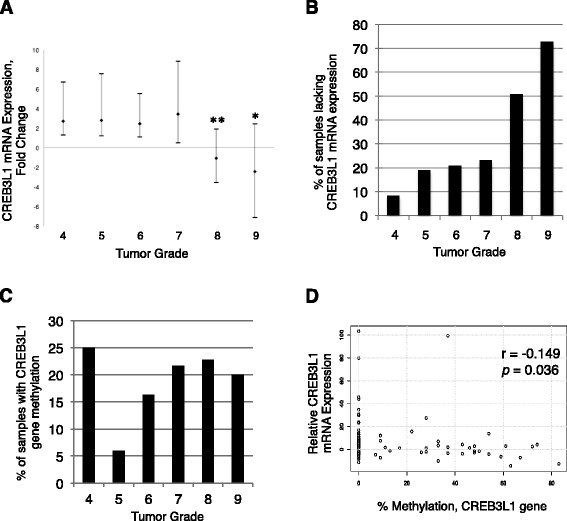
CREB3L1 expression is reduced in high-grade human breast tumor samples. **a** CREB3L1 expression (mRNA) was determined from 213 human breast tumor tissue samples (qPCR) and plotted as fold change relative to the mean value from four normal breast tissue samples (defined as *zero* on the scale), for each tumor grade. Median ± one quartile (numbers of samples for each grade 4–9: 12, 53, 48, 26, 63, 11): *p <0.05, as compared to grades 4–7; **p <0.01, as compared to grades 4–7. **b** A large percentage of the high-grade (8 and 9) tumors lacked CREB3L1 mRNA expression (51 % and 73 %, respectively). An average of 53 % of high-grade breast tumors (8 and 9) are CREB3L1-deficient. **c** *CREB3L1* gene methylation was determined for 201 human breast tumor tissue samples, and was plotted for each tumor according to grade (numbers of samples for each grade 4–9: 12, 50, 49, 23, 57, 10). **d** Negative correlation of CREB3L1 mRNA expression and the level of *CREB3L1* DNA methylation (within nucleotides −15 to 238, relative to the translational start site) in 198 breast cancer tumor samples. Spearman correlation (*r* value) and significance *p* value

We measured CREB3L1 mRNA expression levels by qPCR in 213 tumor samples and found that low- and medium-grade tumors had increased CREB3L1 mRNA expression when compared to normal breast tissue samples (Fig. 5a). In contrast, high-grade breast tumors had reduced CREB3L1 expression. The majority of grade 8 (51 %) and grade 9 (73 %) breast tumors lacked CREB3L1 mRNA expression (Fig. 5b). Overall, CREB3L1 mRNA expression was negatively correlated with tumor type (*r* = −0.325, *p* <0.00001) and tumor grade (*r* = −0.342, *p* <0.00001) (Table 1). In addition, CREB3L1 mRNA expression was positively correlated with age at diagnosis (*r* = 0.294, *p* <0.00001), estrogen receptor expression (*r* = 0.232, *p* <0.001) and weakly with progesterone receptor expression (*r* = 0.158, *p* <0.05). These results suggest that loss of CREB3L1 is more frequently seen in high-grade, more aggressive breast cancers that lack ER and PR expression.

**Table 1:** Clinico-pathological parameters of 213 human breast tumor specimens in relation to CREB3L1 mRNA expression

	CREB3L1 mRNA expression^b^				
	Number^a^	Low	High	*P* value^c^	Spearman *r*
Parameter:					
Age at diagnosis					
<68 years	109	45	64	**0.000013**	0.294
≥68 years	104	25	79		
Tumor type					
Infiltrating ductile	170	66	104	**0.000001**	−0.325
Infiltrating colloid	22	0	22		
Infiltrating lobular	20	2	18		
Infiltrating papillary	1	1	0		
Tumor grade^d^					
Grade 4	12	1	11	**<0.000001**	−0.342
Grade 5	53	11	42		
Grade 6	48	11	37		
Grade 7	26	6	20		
Grade 8	63	32	31		
Grade 9	11	8	3		
Estrogen receptor					
Negative	20	14	6	**0.00064**	0.232
Positive	193	55	138		
Progesterone receptor					
Negative	66	29	37	**0.021**	0.158
Positive	147	40	107		
Lymph node status					
Negative	69	23	46	0.120	−0.120
Positive	101	38	63		
Unknown	43	8	35		

^a^Only patients with primary breast cancer were included. ^b^Based on quantitative real-time PCR results. ^c^Significance of Spearman rank correlation. Significant *p* values are marked in boldface. ^d^Graded according to the Nottingham derivation of the Scarff Bloom Richardson system

Our breast cancer cell line data suggested that CREB3L1 expression can be regulated in part by DNA methylation. Therefore, we also analyzed *CREB3L1* methylation in a large group of breast tumor samples using the same approach we described above for the breast cancer cell lines. Again, this provided qualitative methylation data for 60 CpG sites between −429 and +259 (Additional file 7: Table S3). These tumor samples generally showed less *CREB3L1* DNA methylation than the cell lines had, and similar to the cell line data most methylation was clustered within the 3′ end of the region analyzed. Therefore, midway through our analysis we narrowed our focus to the 24 CpG sites between −15 and +259 for about half of these samples (Additional file 7: Table S3). In all we analyzed 201 tumor samples and found 35 contained CREB3L1 methylation. Tumor samples with fewer methylated sites showed site-specific methylation at the most 3′ end including sites 221, 238 and 259 (Additional file 7: Table S3). These results suggest a hierarchy with the sites at the 3′ end of this −15 to +259 nucleotide region being methylated preferentially to those upstream.

We observed one tumor that showed *CREB3L1* DNA methylation and had relatively high CREB3L1 mRNA levels, prompting us to examine *CREB3L1* gene copy number. According to the Catalogue of Somatic Mutations in Cancer (COSMIC) database, *CREB3L1* is rarely mutated in cancers but shows reduced copy number in 18 % (140/782) of breast cancers. In our samples there were only a few breast tumors with alterations in *CREB3L1* copy number; 9 % had increased copy number (3–5 copies), 4 % had a decreased copy number of 1 and 86 % had a normal copy number of 2. The tumor with *CREB3L1* DNA methylation but high CREB3L1 mRNA levels had a normal gene copy number (data not shown).

We observed that *CREB3L1* DNA methylation was found in some samples within all tumor grades (Fig. 5c). The high methylation frequency observed for grade 4 (3/12 = 25 %) and grade 9 (2/10 = 20 %) were likely significantly influenced by the relatively small sample sizes for these two groups. The sample sizes within the remaining tumor grades (5–8) were larger, making these data more robust. Overall, *CREB3L1* methylation was weakly, positively correlated with tumor type (*r* = 0.150, *p* <0.05) and tumor grade (*r* = 0.146, *p* <0.05) (Table 2). In addition, *CREB3L1* methylation was weakly, negatively correlated with age of diagnosis (*r* = −0.166, *p* <0.05), ER expression (*r* = −0.168, *p* <0.05) and PR expression (*r* = −0.143, *p* <0.05). Our data also showed weak negative correlation between CREB3L1 mRNA expression and *CREB3L1 *methylation (*r* = −0.149, *p* <0.036) (Fig. 5d). These results suggest that the majority of the breast tumor samples with DNA methylation had reduced CREB3L1 mRNA expression, implicating methylation as a mechanism of *CREB3L1* gene silencing.

**Table 2:** Clinico-pathological parameters of 201 human breast tumor specimens in relation to *CREB3L1* gene methylation

	CREB3L1 methylation^b^				
	Number^a^	Low	High	*P* value^c^	Spearman *r*
Parameter:					
Age at diagnosis					
<68 years	100	79	21	**0.018**	−0.166
≥68 years	101	89	12		
Tumor type					
Infiltrating ductile	149	127	22	**0.034**	0.150
Infiltrating colloid	21	21	0		
Infiltrating lobular	29	18	11		
Infiltrating papillary	1	1	0		
Infiltrating tubular	1	1	0		
Tumor grade^d^					
Grade 4	12	9	3	**0.038**	0.146
Grade 5	50	48	2		
Grade 6	49	41	8		
Grade 7	23	18	5		
Grade 8	57	44	13		
Grade 9	10	8	2		
Estrogen receptor					
Negative	14	11	3	**0.017**	−0.168
Positive	187	157	30		
Progesterone receptor					
Negative	54	42	12	**0.042**	−0.143
Positive	147	126	21		
Lymph node status					
Negative	65	52	13	0.532	−0.050
Positive	93	77	16		
Unknown	43	39	4		

^a^Only patients with primary breast cancer were included. ^b^Based on DNA sequencing results with and without sodium bisulfite treatment. ^c^Significance of Spearman rank correlation. Significant *p* values are marked in boldface. ^d^Graded according to the Nottingham derivation of the Scarff Bloom Richardson system

### Larger datasets support a role for *CREB3L1* DNA methylation in the regulation of CREB3L1 expression

**Fig. 6:**
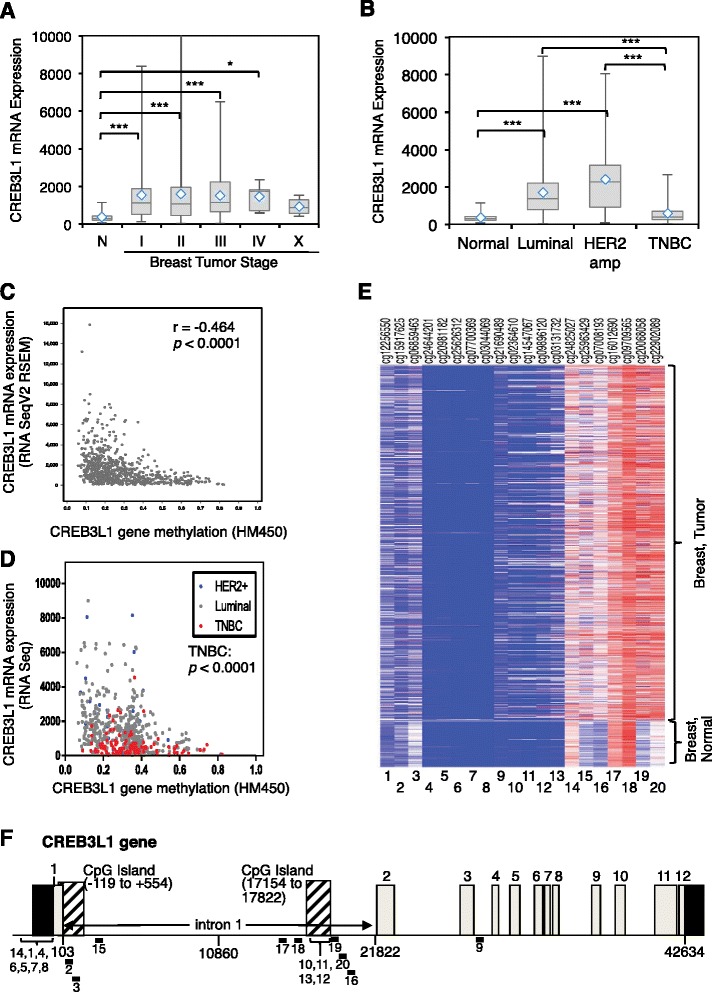
CREB3L1 mRNA expression and DNA methylation from human breast tumor samples in The Cancer Genome Atlas (TCGA) database. **a**, **b** CREB3L1 mRNA expression varies for different tumor stages (**a**: n = normal (70 samples); numbers of samples for each stage I to X: 80, 287, 139, 5, 4) and subtypes (**b**: numbers of samples: 70, 263, 15, 76) of breast tumors. Statistical differences were analyzed using post-hoc pairwise comparison: *p <0.05; ***p <0.001. **c** Using cBioPortal containing TCGA data from 737 breast cancer cases, there is an inverse relationship between CREB3L1 mRNA expression and *CREB3L1* gene methylation that is very significant (Spearman correlation, *r* = −0.464, *p* <0.001). **d** Focusing on samples where the tumor subtype was known, the lowest CREB3L1 mRNA expression and also the highest *CREB3L1* gene methylation was found most often in triple negative breast cancer (TNBC) (*p* <0.0001). **e** Data within TCGA contained an analysis of 26 regions (50 nucleotides each) within the *CREB3L1* gene that had been analyzed for DNA methylation (Additional file 8: Table S4). Of these, 20 showed methylation changes between normal (97 samples) and breast tumors (764 samples), as shown (heat map): *red* high methylation, *white* mean methylation, *blue* low methylation. Methylation regions are numbered (*bottom* (*1–20*)) with the sequences listed in Additional file 8: Table S4, and their approximate locations are shown (**f**). **f** Schematic of CREB3L1 gene organization. The 5′ and 3′ untranslated regions (*black boxes*), exons (*gray boxes* with *numbers* above) and CpG islands (*hatched boxes*) are shown. The first CpG island is 672 nucleotides long between −119 and 554, relative to the translational start site, and contains 51 CpG sites. The second CpG island is 610 nucleotides long between 17154 and 17822 within intron 1 and contains 56 CpG sites. The approximate locations of 20 cg probes for methylation are indicated underneath and numbered as in **e**

We then expanded our analysis of CREB3L1 mRNA expression and methylation to the large TCGA dataset. CREB3L1 mRNA levels were increased in breast tumors, particularly for early-stage tumors (Fig. 6a). Similar to what we had observed for our smaller tumor dataset, CREB3L1 mRNA expression was increased in luminal and HER2 breast cancers but was low in TNBC (Fig. 6b). CREB3L1 mRNA expression also inversely correlated with *CREB3L1* DNA methylation as noted above (Fig. 6c, cBioPortal: *r* = −0.464, *p* <0.0001). TNBCs were found to have both the lowest CREB3L1 mRNA levels and the highest *CREB3L1* DNA methylation (Fig. 6d, *p* <0.0001), suggesting the low CREB3L1 often observed in TNBC cells may in part be due to increased methylation.

The methylation analysis carried out for these samples included assessing 26 50- nucleotide regions, each containing one or more CpG sites within the *CREB3L1* gene (Additional file 8: Table S4). Five of these regions did not contain any methylation, and one had methylation but it was unchanged in normal and tumor breast samples. The remaining 20 methylation regions were differentially methylated between normal breast and breast tumor samples (Fig. 6e). We assigned each of these 20 regions a number from 1–20 for easy reference and show the location of each region that was analyzed, relative to the overall gene structure for *CREB3L1* (Fig. 6f) and their precise CpG-containing sequences for those in the promoter region (Additional file 3: Figure S2). We determined the relative methylation for each region in normal versus breast tumor samples to assess which regions might be involved in regulating CREB3L1 expression changes (Additional file 9: Figure S5a, b). Many of the tested regions did not change their methylation status appreciably, including those in the 5′ UTR (numbers 14, 1, 4, 6, 5, 7, and 8), some within the 20 kb intron 1 (numbers 15, 17, and 18), those within a second CpG island within intron 1 (numbers 10, 11, 13, and 12), or within intron 3 (number 9). We focused on the remaining five regions (numbers 2, 3, 16, 19, and 20). We found that methylation of two regions (numbers 2 and 3), within the first CpG island, negatively correlated with CREB3L1 mRNA expression (Additional file 9: Figure S5c), and they were less methylated in breast tumor samples (Additional file 9: Figure S5a). The remaining three regions (numbers 16, 19, and 20) are all located in a *shore* near the second CpG island (Fig. 6f). Methylation in each of these regions positively correlated with CREB3L1 mRNA expression (Additional file 9: Figure S5d) and all were significantly more methylated in breast tumor samples compared to normal breast tissue (Additional file 9: Figure S5b). The inverse relationship between methylation at regions number 2 and 3, coupled with the direct relationship between methylation at regions number 16, 19, and 20 raises the possibility that complex methylation-dependent changes at several regions within the *CREB3L1* gene (Additional file 10: Figure S6) could contribute to the regulation of CREB3L1 expression (Fig. 6b). When subdivided into low and high methylation, as compared to the median methylation of the normal breast samples, all five regions showed a significant difference in the relative CREB3L1 mRNA expression for the two groups (Additional file 11: Figure S7). Region number 2 showed the largest significant difference in methylation between normal breast and breast tumors, suggesting that changes in methylation in this region may have the largest influence on CREB3L1 mRNA expression. Region number 2 contains two CpG sites at nucleotides 259 and 238 (Additional file 3: Figure S2), the most frequently methylated within the breast tumor cell lines and breast tumor samples that we analyzed within our datasets (Additional file 5: Tables S2 and Additional file 7: Table S3).

### Poor prognosis for patients with low CREB3L1 mRNA expression in luminal A breast cancer and TNBC

**Fig. 7:**
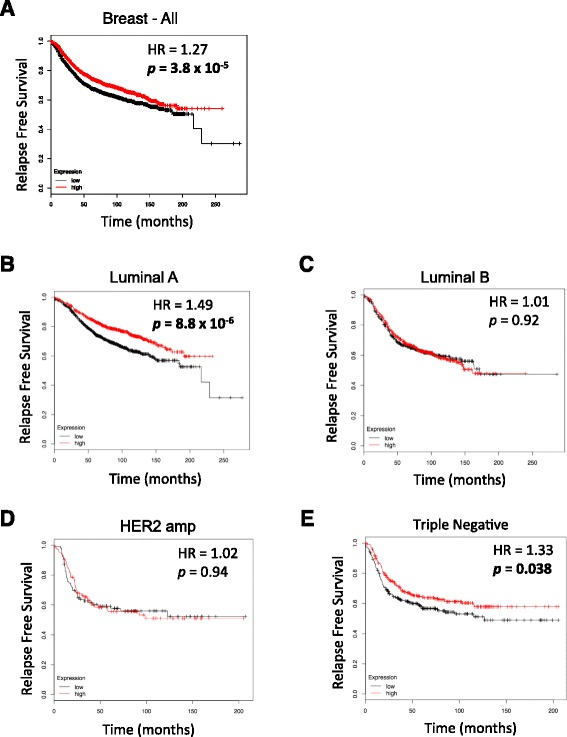
Poorer prognosis for luminal A breast cancer and triple negative breast cancer (TNBC) patients is associated with low expression of CREB3L1. Kaplan–Meier relapse-free survival probability for breast cancer patients with high (*red*) and low (*black*) CREB3L1 gene expression (divided at the median). The online software, KM plotter, was used to access gene expression data and relapse-free survival information from Gene Expression Omnibus (Affymetrix microarrays only), European Genome-phenome Archive and The Cancer Genome Atlas. The two patient cohorts were compared and the hazard ratio (HR) with 95 % confidence intervals and log-rank *p* value were calculated for the low versus high CREB3L1 levels. Relapse-free survival probability for breast cancer patients: all (**a**; *n* = 3554), luminal A (**b**; n = 1764), luminal B (**c**; n = 1002), human epidermal growth factor receptor 2 (*HER2*) amplified (**d**; n = 208) and triple negative (**e**; n = 580) divided into low or high CREB3L1 mRNA expression groups

Kaplan–Meier survival analysis was performed using KM-plotter [43] with gene expression data and relapse-free survival information accessed from several sources including GEO (Affymetrix microarrays only), EGA and TCGA (Fig. 7). When all breast cancers were analyzed as a group there was a shorter relapse-ree survival time for patients whose tumors contained low levels of CREB3L1 mRNA expression (hazard ratio (HR) = 1.27; *p* <0.0001) (Fig. 7a). When breast cancers were subdivided into different molecular classifications, CREB3L1 mRNA expression did not significantly influence the relapse-free survival time for luminal B (ER+ and/or PR+, HER+ or HER2– with high Ki67) (Fig. 7c) and HER2 (Fig. 7d) breast cancer patients. In contrast, both luminal A (ER+ and/or PR+, HER2–, low Ki67) (Fig. 7b) and triple negative (Fig. 7e) breast cancer patients with low CREB3L1 mRNA expression had higher HRs (1.49 and 1.33, respectively) that were statistically significant (*p* <0.00001 and *p* <0.05, respectively). These results strongly implicate the loss of CREB3L1 expression as an indicator of poor prognosis in breast cancers that are HER2–, such as luminal A and triple negative.

**Fig. 8:**
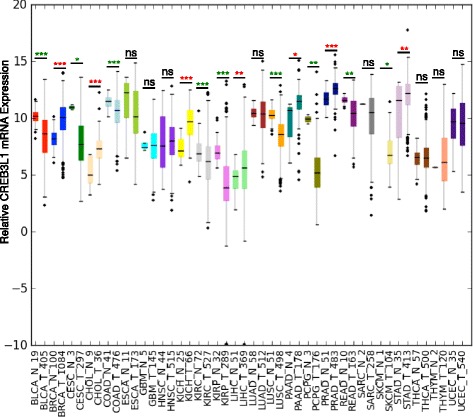
Differential CREB3L1 mRNA expression between normal and tumor tissue for 48 human cancer types. Data obtained from The Cancer Genome Atlas database, with the number of patients in each group indicated on the *x-axis*: median ± one quartile; *p <0.05; **p <0.01; ***p <0.001; *ns* not significant. Cancer tissues with CREB3L1 expression higher in tumor (*T*) than normal (*N*) (*red asterisks*) include: *BRCA* breast carcinoma, *PRAD* prostate adenocarcinoma, *KICH* chromophobe renal cell carcinoma, *CHOL* cholangiocarcinoma, *LIHC* liver hepatocellular carcinoma, *STAD* stomach adenocarcinoma, *PAAD* pancreatic ductal carcinoma. Cancer tissue with CREB3L1 expression lower in tumor than normal (*green asterisks*) include: *LUSC* lung squamous cell carcinoma, *KIRC* clear cell kidney carcinoma, *KIRP* papillary kidney carcinoma, *BLCA* bladder urothelial carcinoma, *COAD* colon adenocarcinoma1, *PCPG* pheochromocytoma and paraganglioma, *READ* rectal adenocarcinoma, *SKCM* cutaneous melanoma, *CESC* cervical squamous cell carcinoma. Normal and tumor tissues with similar CREB3L1 expression include *ESCA* esophageal cancer, *GBM* glioblastoma multiforme, *HNSC* head and neck squamous cell carcinoma, *LUAD* lung adenocarcinoma, *SARC* sarcoma, *THCA* papillary thyroid carcinoma*, THYM* thymoma, *UCEC* uterine corpus endometrial carcinoma

Using the TCGA database, we determined if CREB3L1 mRNA expression was differentially regulated between normal tissues and the corresponding tumor tissues (Fig. 8). Several tumor tissues had little or no change in CREB3L1 mRNA expression as compared to normal tissue, including esophageal cancer, glioblastoma multiforme, head and neck squamous cell carcinoma, lung adenocarcinoma, sarcoma, papillary thyroid carcinoma, thymoma and uterine corpus endometrial carcinoma. A second group of cancers had significantly higher CREB3L1 mRNA expression than their normal counterparts, including breast carcinoma (*p* = 6.5 × 10^−31^), prostate adenocarcinoma (*p* = 1.5 × 10^−11^), chromophobe renal cell carcinoma (*p* = 1.9 × 10^−9^), cholangiocarcinoma (*p* = 5.5 × 10^−4^), stomach adenocarcinoma (*p* = 0.0025), liver hepatocellular carcinoma (*p* = 0.0027) and pancreatic ductal carcinoma (*p* = 0.019). Although these cancer types (including breast cancer) typically show an increase in CREB3L1 expression, patients with tumors expressing low levels of CREB3L1 may experience a poorer prognosis, as we found for breast cancer (Fig. 7a). A third group of cancers had significantly lower CREB3L1 mRNA expression than the corresponding normal tissues, including: lung squamous cell carcinoma (*p* = 1.7 × 10^−19^), clear cell kidney carcinoma (*p* = 1.2 × 10^−10^), papillary kidney carcinoma (*p* = 1.2 × 10^−10^), bladder urothelial carcinoma (*p* = 2.8 × 10^−6^), colon adenocarcinoma (*p* = 0.00010), pheochromocytoma and paraganglioma (*p* = 0.0029), rectal adenocarcinoma (*p* = 0.0057), cutaneous melanoma (*p* = 0.043) and cervical squamous cell carcinoma (*p* = 0.046). Thus, the loss of CREB3L1 is a frequent occurrence and could play an important role in tumor progression and metastasis in many cancer types.

## Discussion

Previous investigations by our laboratory have implicated *CREB3L1* as a metastatic suppressor gene in breast cancer models in vitro and in vivo. Transfection of CREB3L1 into cells that normally express low levels of CREB3L1 reduced cell migration, invasion, anchorage-independent growth and tolerance of hypoxia [25]. Consistent with these effects, knockdown of endogenous CREB3L1 in cells demonstrated that loss of CREB3L1 expression significantly increased migration, invasion, anchorage-independent growth, and tolerance of hypoxia. Further support for a role for CREB3L1 as a metastatic suppressor was provided by in vivo studies where rats were injected with CREB3L1-null or CREB3L1-expressing cells. The CREB3L1-null cells formed large primary tumors (29/30 animals) with frequent lymph node metastases (26/30 animals) [25]. Strikingly, the CREB3L1-expressing cells failed to develop metastases (0/30 animals) and after initially forming large tumors (>0.5 cm^3^), 70 % of these tumors (21/30) regressed to a nearly undetectable size [25].

Here we have expanded upon our previous study to include 40 human breast cancer cell lines and over 200 human breast cancer tumor samples and investigated DNA methylation and its role in the regulation of CREB3L1 expression. We found that in luminal and HER2 amplified breast cell lines and tumors, CREB3L1 mRNA expression was frequently increased, whereas in TNBC cell lines and tumor samples, CREB3L1 expression was frequently low. As CREB3L1 has an important role in metastasis suppression, its low expression in TNBC may contribute to its more aggressive and metastatic phenotype.

CREB3L1 is activated in response to cellular stress as part of the endoplasmic reticulum stress response process [53–55]. Viral infections and treatment with the chemotherapy agent, doxorubicin, have also been shown to induce cell stress and the activation of CREB3L1 [21, 24]. Tumors form in a stressful cellular environment with low nutrients and low oxygen levels (hypoxia), suggesting that CREB3L1 may be activated during tumor development and progression.

A recent study demonstrated that CREB3L1 expression was required for the chemotherapeutic agent doxorubicin to block the proliferation of cancer cells [21]. Doxorubicin, but not other chemotherapy agents that cause DNA damage (etoposide, bleomycin) or cell cycle arrest (paclitaxel), cause increased ceramide production that induces the trafficking of CREB3L1 from the endoplasmic reticulum to the Golgi complex [21]. CREB3L1 has been shown to be proteolytically activated and the mature protein translocated into the nucleus where it could alter the transcription of genes important for cell proliferation [21, 24]. As doxorubicin was only effective in blocking cell proliferation in CREB3L1-expressing cells, this suggests that only patients expressing CREB3L1 are likely to benefit from doxorubicin treatment. In support of this idea, a recent report showed that higher levels of CREB3L1 expression strongly inversely correlated with tumor volume upon doxorubicin treatment in renal cell carcinoma xenografts (*r* = −0.891; *p* = 0.017) [22], leading to the suggestion that CREB3L1 could be a biomarker that predicts doxorubicin treatment outcome. These results also suggest that the loss of CREB3L1 may contribute to doxorubicin treatment resistance.

Other members of the UPR have been implicated in tumor development. GRP78 has been shown to protect tumor cells from cytotoxic T cell immune response and apoptosis following Ca^2+^ depletion [56]. Additionally, increased GRP78 expression has been associated with chemoresistance in breast cancer [15]. PERK and XBP1 are both important factors in tolerance of hypoxia; loss of expression of either factor inhibits tumor growth and increased apoptosis following hypoxia [57, 58]. Conversely, decreased expression of UPR transducers has been observed in the progression from normal to high-grade tumors in mouse models of prostate cancer [53, 55]. It is thought that increased activation of the UPR may impart tolerance of hypoxia in some tumors, and downregulation may promote tumor progression by preventing activation of apoptosis [55].

We have assessed human breast tumor samples of various grades for the expression of CREB3L1. Low-grade and medium-grade breast tumors had increased CREB3L1 expression, when compared to normal breast tissue samples. In contrast, high-grade (8 and 9) breast tumors had reduced CREB3L1 expression (*p* = 0.001). Our results suggest that CREB3L1 expression is initially upregulated in response to the stressful conditions that exist within the tumor environment, as observed for other stress response proteins [53–55]. In contrast to other stress response proteins, loss of CREB3L1 expression is prevalent in high-grade tumors and may be required to avoid apoptosis under prolonged stress conditions. This would allow the de-repression of genes necessary for angiogenesis and metastasis, which we have shown are negatively regulated by CREB3L1 [25]. Overall, CREB3L1 was lost in 31 % (67/213) of the human breast tumor samples analyzed, but importantly CREB3L1 was lost from a much larger fraction of the high-grade (8 and 9) metastatic breast tumors (51 % of grade 8 and 73 % of grade 9 breast tumors; *p* = 0.001). Thus, CREB3L1 may provide a cytoprotective effect early in tumor development and later decreased expression allows progression to high-grade tumors.

Our analysis also found that breast cancer patients with low CREB3L1 expression have a shorter relapse-free survival time specifically for the luminal A and TNBC subtypes. As similar results were not seen for luminal B and HER2 amplified breast cancers, the impact of low CREB3L1 expression may not be significant in the context of HER2 expression. This suggests that low CREB3L1 is a marker for poor prognosis in both luminal A breast cancer and TNBC.

Our data in human breast cancer cell lines suggest that epigenetic silencing of CREB3L1 contributes to reducing CREB3L1 mRNA expression for at least some cell lines, an effect that was reversed by the inhibition of histone acetylation with TSA and/or inhibition of DNA methylation with DAC. We also found that some cell lines had little or no *CREB3L1* DNA methylation and yet, still had little or no CREB3L1 mRNA. As methylation outside of the region tested could also impact *CREB3L1* transcription it is possible that other regions within the *CREB3L1* gene also have key roles in its regulation. In this regard the analysis of TCGA data for *CREB3L1* DNA methylation suggested there might be additional CpG sites outside of the 688 nucleotides tested that could influence CREB3L1 mRNA expression. For example, regions number 16, 19 and 20 (near the 3′ end of intron 1) all had increased methylation in breast tumor samples as compared to normal breast tissue (Additional file 9: Figure S5b), raising the possibility that methylation in these regions could influence CREB3L1 mRNA expression.

In contrast, some cell lines had significant levels of *CREB3L1* DNA methylation, yet expressed relatively high levels of CREB3L1 mRNA (e.g., MDA-MB-415, MDA-MB-231). This could also be due to changes in methylation within other regions of the *CREB3L1* gene that could impact transcription. In addition to DNA methylation, CREB3L1 mRNA levels can also be influenced by alterations in histone modifications, as well as by the regulation of RNA processing, transport and stability [59, 60]. Further the high CREB3L1 mRNA levels and low protein expression observed in some breast cancer cell lines (e.g., HCC202) could be the result of translational deregulation or the rapid turnover of CREB3L1 protein. An E3 ubiquitin ligase, HRD1, has been reported to ubiquitinate CREB3L1 to induce proteasomal-mediated degradation in C6 glioma cells and mouse embryonic fibroblasts to maintain low levels of CREB3L1 protein [52]. Other cell lines have an abundance of CREB3L1 protein, but very low levels of the corresponding mRNA (e.g., HCC1500), raising the possibility that the protein is not being turned over as rapidly in these cells, perhaps as a result of defects in HRD1-mediated ubiquitination and degradation.

Within the region tested (−429 to +259, numbering relative to the translational start site), we noted that for cell lines with less *CREB3L1* DNA methylation, the most frequently methylated CpG sites were 259 and 238. Cell lines with more methylation had an increased number of methylated CpG sites 5′ to these, suggesting that *CREB3L1* DNA methylation proceeds in a hierarchical fashion with some sites methylated preferentially. Although the human breast tumor samples typically had lower levels of *CREB3L1* DNA methylation, they also had similar preferential methylation at CpG sites 221, 238 and 259. The analysis of *CREB3L1* DNA methylation in breast tumors in the TCGA database, also with suggested CpG sites 238 and 259 (i.e., region number 2), were usually more methylated in tumors with low CREB3L1 mRNA expression (Additional file 11: Figure S7), but were generally less methylated in breast tumors than in normal breast tissue (Additional file 9: Figure S5a). Together these results suggest that *CREB3L1* methylation at these sites in particular may negatively regulate CREB3L1 expression.

Overall, CREB3L1 expression was negatively correlated with its gene methylation, suggesting that epigenetic silencing is one mechanism that contributes to decreased CREB3L1 levels. Increased *CREB3L1* gene methylation and low CREB3L1 mRNA expression were both correlated with more aggressive types of breast cancer [61] and higher tumor grade (8 and 9). We therefore conclude that the loss of CREB3L1 expression in cells of high metastatic potential is due in some cases to methylation of a region of CpG sites near the CREB3L1 start site. In support of this, high levels of *CREB3L1* DNA methylation and low levels of CREB3L1 mRNA expression were more frequently observed in TNBC cell lines and breast tumor samples. This is consistent with previous reports showing more DNA hypermethylation in TNBC [62], in some cases due to overexpression of DNA methyltransferase enzymes [63].

The subcellular localization of CREB3L1 also changes with tumor progression. In low-grade breast tumors CREB3L1 is predominantly a nuclear protein, consistent with its processing into the mature form and translocation into the nucleus as part of the cellular stress response to activate CREB3L1. High-grade breast tumors that still express CREB3L1 typically have mainly cytoplasmic protein localization, which likely would prevent CREB3L1-mediated transcriptional repression of target genes that promote cell growth, survival, migration, invasion, angiogenesis and metastasis [25]. Similar subcellular localization patterns of CREB3L1 are observed during bladder cancer progression, suggesting a common function for CREB3L1 in at least these two cancer types [20].

Our results in TNBC and high-grade tumors are in agreement with a recent study by Rose et al*.* who found that CREB3L1 gene expression was downregulated in bladder cancer and that the loss of expression was associated with DNA methylation of the gene [20]. They also reported that DNA methylation was associated with invasive tumor subtypes of bladder cancer. In support of a wider role for CREB3L1 in multiple cancer types, we determined that CREB3L1 mRNA expression is altered in many types of cancer (Fig. 8). Cancers of the breast, prostate, kidney (papillary), stomach and pancreas all had generally increased CREB3L1 expression in tumors as compared to the corresponding normal tissue. Our work in breast cancer suggests that this should provide a protective effect. However, as we saw for breast cancers with reduced CREB3L1, like TNBC, the disease is typically more aggressive and advanced resulting in a poor prognosis within these groups if tumors have reduced CREB3L1. Importantly, we identified a large group of cancer types where low CREB3L1 expression is prevalent, including lung squamous cell carcinoma, melanoma and cancers of the kidney (clear cell), bladder, colon, liver, adrenal gland, rectum and cervix. This raises the possibility that loss of CREB3L1 could play a key role in cancer progression and metastasis across a broad group of cancer types.

## Conclusions

In conclusion, we have demonstrated that CREB3L1 is frequently upregulated in luminal and HER2 amplified breast cancer, but not in TNBC. Tumors in TNBC are known to have hypermethylated DNA and consistent with this, we see enhanced methylation of several key CpG sites within the *CREB3L1* gene that strongly correlate with reduced expression in breast cancer cell lines and in human breast tumors. Low CREB3L1 expression is strongly associated with more aggressive, high-grade tumors. Our results also show that reduced CREB3L1 mRNA is an indicator of poor prognosis, specifically in luminal A breast cancer and TNBC. The discovery that CREB3L1 expression is frequently altered in many cancer types suggests it could have a broad role in cancer progression and metastasis. In addition, loss of CREB3L1 expression could have prognostic value for multiple forms of cancer.

## Additional files

Additional file 1: Table S1. Primers for *CREB3L1* gene sequencing to determine methylation. (PDF 28 kb) Additional file 2: Figure S1. CREB3L1 protein expression in a panel of breast cancer cell lines. Cell lysates (50 μg protein/lane) from the indicated breast cancer cell lines were probed for CREB3L1 and β-actin (loading control). The full-length precursor form (approximately 84 kDa) and processed mature form (56–58 kDa) of CREB3L1 are indicated (*arrows*). A control lysate from HCC1806 cells transfected with HA-tagged CREB3L1 was also included (HA-CREB3L1). It has both the precursor (*P*) and cleaved, mature (*M*) forms of CREB3L1, and as a result of the triple HA-tag, they are slightly larger in size (approximately 6 kDa) than the endogenous CREB3L1 proteins. *Background band. (PDF 213 kb) Additional file 3: Figure S2. CREB3L1 promoter region nucleotide sequence with locations of CpG sites. Non-coding nucleotides (*lower case*) and coding nucleotides (*upper case*) are shown. CpG sites are highlighted (*red* and *blue*). The transcriptional start site (*TSS*) and the translational start site (coding region) are indicated. The numbering is relative to the translational start site (+1). The CpG island (−118 to +554), as determined using the USCS genome browser, is located within the *boxed* and *shaded* region. For the analysis of methylation in breast tumor samples from the The Cancer Genome Atlas database the location of the various cg probes are numbered, labeled and indicated with *lines* either *below* or *above* the sequence. (PDF 48 kb) Additional file 4: Figure S3. Analysis of CREB3L1 DNA methylation for 40 breast cancer cell lines. **a** Schematic diagram of the CREB3L1 promoter region. The 5′ untranslated region (*5′UTR*; *black box*), the coding region (*gray box*) and CpG island (*hatched box*) and the exon and intron boundaries are indicated. **b** Sixty CpG sites were evaluated for their methylation between −429 and +259, relative to the translational start site. The frequency of methylation at each site is indicated. Samples with less methylation, invariably had the more 3′ CpG sites methylated preferentially (i.e., 259 and 238) and as more methylation was observed additional CpG sites were methylated towards the 5′ end (as evident in Additional file 5: Table S2). **c** Examples of the scoring system used for methylation. Predominantly methylated sites had a majority of *C* residues protected from sodium bisulfite conversion and were assigned a value of 2. Somewhat methylated sites had protected *C* nucleotides above background levels, but less than the predominant *T* at the same site, and were assigned a value of 1. Unmethylated CpG sites were scored as 0. (PDF 149 kb) Additional file 5: Table S2. Site-specific methylation data for a panel of 40 breast cancer and 4 non-tumorigenic breast cell lines. (PDF 58 kb) Additional file 6: Figure S4. CREB3L1 protein expression and subcellular localization for each tumor subtype. CREB3L1 protein expression and subcellular localization were determined for 97 human tumor samples and 41 were found to have CREB3L1. **a** Luminal and human epidermal growth factor receptor 2 (*HER2*) amplified breast tumors had a similar proportion of tumors expressing and lacking CREB3L1 protein. **b** HER2 amplified tumors had a higher proportion of cytoplasmic CREB3L1 as compared to nuclear CREB3L1 when CREB3L1 protein was present. (PDF 40 kb) Additional file 7: Table S3. Site-specific methylation data for the 35 breast tumor samples that contained methylated sites. (PDF 46 kb) Additional file 8: Table S4. Probe information used in The Cancer Genome Atlas (*TCGA*) database to analyze *CREB3L1* gene methylation in human breast tumor samples. (PDF 38 kb) Additional file 9: Figure S5. Methylation in normal and tumor breast tissue within specific CpG regions of the *CREB3L1* gene. The Cancer Genome Atlas (*TCGA*) database contained methylation data for 20 regions within the *CREB3L1* gene (Fig. 6e) for normal and tumor breast samples; the locations of these regions are indicated in Fig. 6f and Additional file 8: Table S4. Some are also shown in more detail in Additional file 3: Figure S2. **a** Methylation of CREB3L1 DNA in regions 2 and 3 are reduced in breast tumor samples, as compared to normal breast tissue, and this negatively correlates with CREB3L1 mRNA expression (**c**). Methylation was significantly different between tumor and normal breast tissue in all regions except number 6: region 1 (*p* = 4.8 × 10^−5^), region 2 (*p* = 1.2 × 10^−9^), region 3 (*p* = 4.8 × 10^−20^), region 4 (*p* = 0.0026), region 5 (*p* = 2.1 × 10^−6^), region 7 (*p* = 1.1 × 10^−7^), region 8 (*p* = 7.6 × 10^−6^), region 9 (*p* = 1.5 × 10^−18^) and region 10 (*p* = 3.1 × 10^−11^). **b** Methylation was significantly different between tumor and normal breast tissue in all regions except number 17: region 11 (*p* = 1.6 × 10^−7^), region 12 (*p* = 1.0 × 10^−9^), region 13 (*p* = 2.3 × 10^−17^), region 14 (*p* = 0.00058), region 15 (*p* = 2.6 × 10^−19^), region 16 (*p* = 1.6 × 10^−45^), region 18 (*p* = 9.7 × 10^−9^), region 19 (*p* = 1.8 × 10^−45^) and region 20 (*p* = 1.1 × 10^−42^). **d** In addition, methylation in regions 16, 19 and 20 increased substantially in breast tumor samples and positively correlated with CREB3L1 mRNA expression. (PDF 294 kb) Additional file 10: Figure S6. Methylation within specific CpG regions of the *CREB3L1* gene in different breast tumor subtypes. The relative methylation was plotted for each tumor subtype. Methylation in regions 2 and 3 show an inverse correlation with CREB3L1 mRNA expression (found in Fig. 6b), whereas methylation in regions 16, 19 and 20 show a direct correlation with CREB3L1 mRNA expression. For all panels: normal (n = 97), luminal (n = 357), human epidermal growth factor receptor 2 (*HER2*) amplified (n = 19), triple negative breast cancer (*TNBC*) (n = 113). Statistical differences were analyzed using post-hoc pairwise comparison: **p* <0.05; ***p* <0.01; ****p* <0.001. (PDF 97 kb) Additional file 11: Figure S7. CREB3L1 mRNA expression varies in breast tumors with *CREB3L1* gene methylation in specific regions. For each of the CpG regions shown, tumor samples were divided into low and high methylation groups based on the level of methylation relative to the median methylation in that region in normal breast tissue and plotted according to the level of CREB3L1 mRNA expression for the corresponding samples. Number of samples in each group: site 2: 418-L, 133-H; site 3: 451-L, 100-H; site 16: 114-L, 437-H; site 19: 32-L, 519-H; site 20: 27-L, 524-H. The median methylation in normal breast tissue was as follows: site 2 (0.2556), site 3 (0.4189), site 16 (0.2708), site 19 (0.3352), and site 20 (0.4669). Methylation was significantly different between tumor and normal breast tissue in all regions: region 2 (*p* = 1.9 × 10^−21^), region 3 (*p* = 4.0 × 10^−20^), region 16 (*p* = 4.6 × 10^−5^), region 19 (*p* = 6.4 × 10^−10^) and region 20 (*p* = 1.1 × 10^−7^). (PDF 39 kb)

## Abbreviations

ATF6: activating transcription factor-6
ATCC: American Type Culture Collection
bp: base pair(s)
CRE: cyclic AMP responsive elements
CREB3L1: cAMP-responsive element-binding protein 3-like protein 1
DAC: 5-aza-2′-deoxycytidine
EGA: European Genome-phenome Archive
ER: estrogen receptor
ESRE: endoplasmic reticulum stress responsive elements
GAPDH: glyceraldehyde-3-phosphate dehydrogenase
GEO: Gene Expression Omnibus
HER2: human epidermal growth factor receptor 2
HR: hazard ratio
IHC: immunohistochemical
IRE1: inositol requiring 1
kDa: kiloDalton
OASIS: old astrocyte specifically-induced substance
PERK: PRK-like endoplasmic reticulum kinase
PR: progesterone receptor
qPCR: quantitative real time PCR
S1P: site-1-protease
TCGA: The Cancer Genome Atlas
TNBC: triple negative breast cancer
TSA: trichostatin A
UPR: unfolded protein response
UTR: untranslated region
XBP1: X-box binding protein 1
