# Supplementary material for: Epigenetic silencing of CREB3L1 by DNA methylation is associated with high-grade metastatic breast cancers with poor prognosis and is prevalent in triple negative breast cancers
Source: Breast Cancer Res. 2016 Jan 25;18:12. doi: 10.1186/s13058-016-0672-x (PMC4727399; doi:10.1186/s13058-016-0672-x)
Supplement: Additional file 1: Table S1. — Primers for CREB3L1 gene sequencing to determine methylation. (PDF 28 kb) [file 13058_2016_672_MOESM1_ESM.pdf]

**Table. S1.**
**Primers for CREB3L1 gene sequencing to determine methylation.**

| 5' Primer | 3' Primer | Fragment Location* | Annealing Temperature (˚C) |
|---|---|---|---|
| TTTGGTTAGTTGGGTTAG TTAGTTAGG | CCCCAAATCCAAAAAA CTAAATC | -492 to +68 | 56 |
| GTTTTGGAGTGAGGGAA TTTAGTG | ATATTCAAAAATTAAAC CCCCAAAA | -51 to +290 | 60 |
| GTTTTGGAGTGAGGGAA GTTTA | CTTAACTCCAAACACA TATC | -51 to +255 | 56 |

*Relative to transcription start site
