# Supplementary figures and images for: Epigenetic silencing of CREB3L1 by DNA methylation is associated with high-grade metastatic breast cancers with poor prognosis and is prevalent in triple negative breast cancers
Source: Breast Cancer Res. 2016 Jan 25;18:12. doi: 10.1186/s13058-016-0672-x (PMC4727399; doi:10.1186/s13058-016-0672-x)

Fig. S1. Ward *et al.*

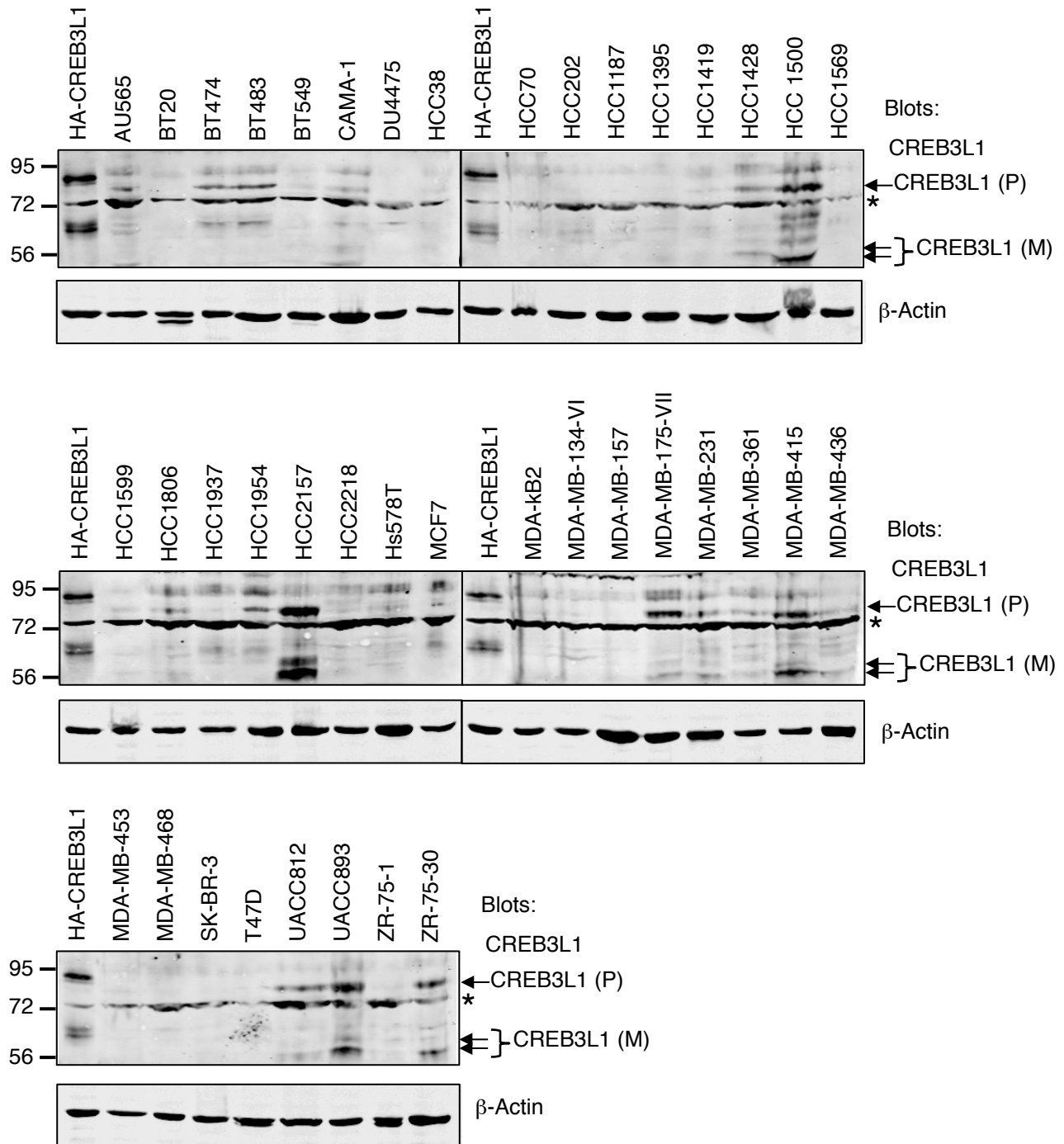

Supplement: Additional file 2: Figure S1. — CREB3L1 protein expression in a panel of breast cancer cell lines. Cell lysates (50 μg protein/lane) from the indicated breast cancer cell lines were probed for CREB3L1 and β-actin (loading control). The full-length precursor form (approximately 84 kDa) and processed mature form (56–58 kDa) of CREB3L1 are indicated (arrows). A control lysate from HCC1806 cells transfected with HA-tagged CREB3L1 was also included (HA-CREB3L1). It has both the precursor (P) and cleaved, mature (M) forms of CREB3L1, and as a result of the triple HA-tag, they are slightly larger in size (approximately 6 kDa) than the endogenous CREB3L1 proteins. *Background band. (PDF 213 kb) [file 13058_2016_672_MOESM2_ESM.pdf]

# **A** CREB3L1 gene

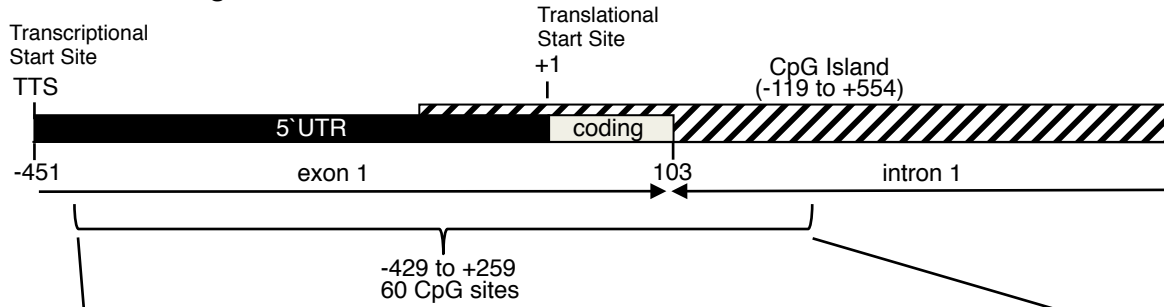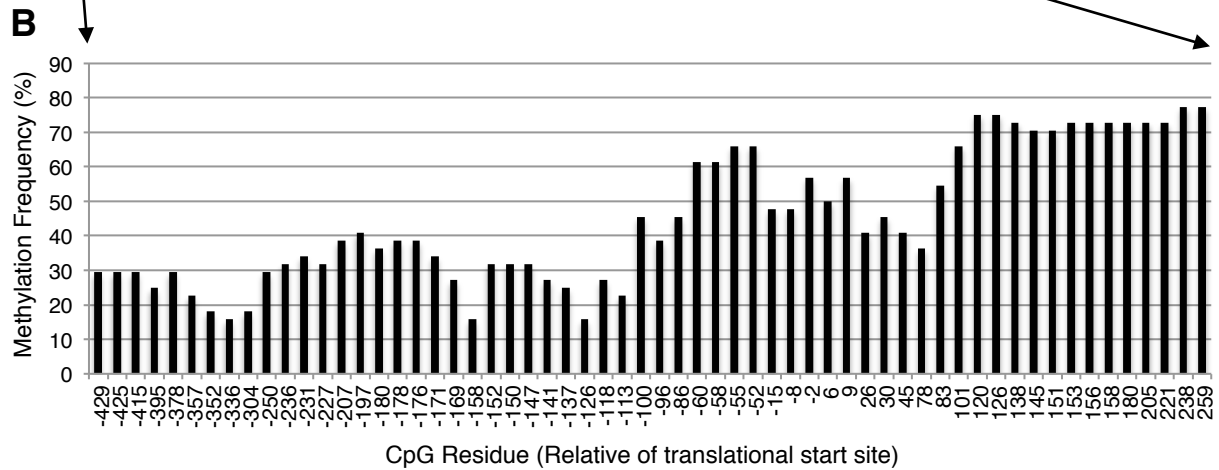

# **C** Examples of Scoring of Methylation Sites:

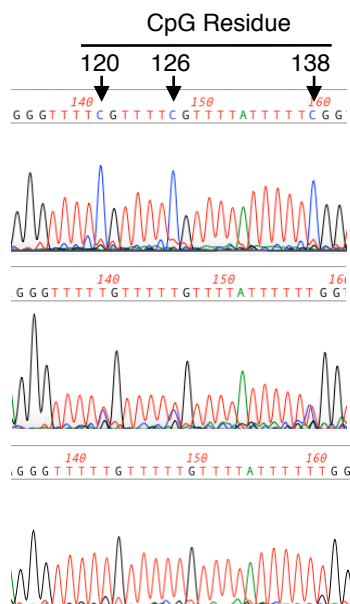

Supplement: Additional file 4: Figure S3. — Analysis of CREB3L1 DNA methylation for 40 breast cancer cell lines. a Schematic diagram of the CREB3L1 promoter region. The 5′ untranslated region (5′UTR; black box), the coding region (gray box) and CpG island (hatched box) and the exon and intron boundaries are indicated. b Sixty CpG sites were evaluated for their methylation between −429 and +259, relative to the translational start site. The frequency of methylation at each site is indicated. Samples with less methylation, invariably had the more 3′ CpG sites methylated preferentially (i.e., 259 and 238) and as more methylation was observed additional CpG sites were methylated towards the 5′ end (as evident in Additional file 5: Table S2). c Examples of the scoring system used for methylation. Predominantly methylated sites had a majority of C residues protected from sodium bisulfite conversion and were assigned a value of 2. Somewhat methylated sites had protected C nucleotides above background levels, but less than the predominant T at the same site, and were assigned a value of 1. Unmethylated CpG sites were scored as 0. (PDF 149 kb) [file 13058_2016_672_MOESM4_ESM.pdf]

Fig. S4. Ward *et al.*

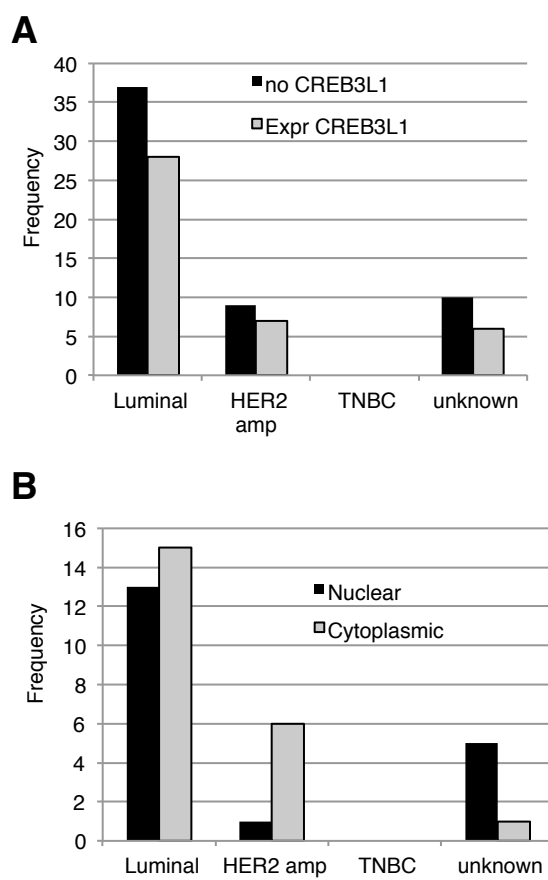

Supplement: Additional file 6: Figure S4. — CREB3L1 protein expression and subcellular localization for each tumor subtype. CREB3L1 protein expression and subcellular localization were determined for 97 human tumor samples and 41 were found to have CREB3L1. a Luminal and human epidermal growth factor receptor 2 (HER2) amplified breast tumors had a similar proportion of tumors expressing and lacking CREB3L1 protein. b HER2 amplified tumors had a higher proportion of cytoplasmic CREB3L1 as compared to nuclear CREB3L1 when CREB3L1 protein was present. (PDF 40 kb) [file 13058_2016_672_MOESM6_ESM.pdf]

Fig. S5. Ward *et al.*

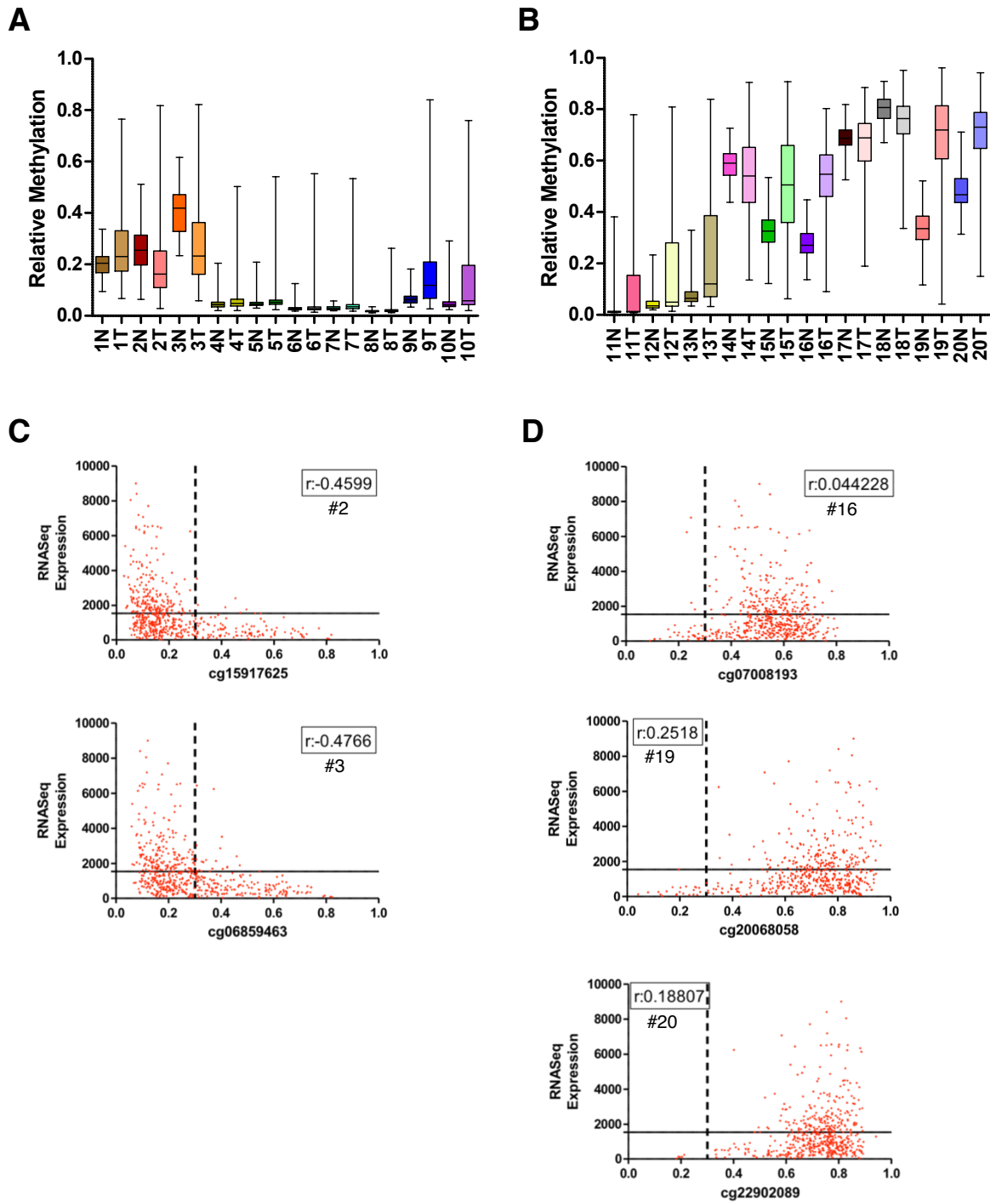

Supplement: Additional file 9: Figure S5. — Methylation in normal and tumor breast tissue within specific CpG regions of the CREB3L1 gene. The Cancer Genome Atlas (TCGA) database contained methylation data for 20 regions within the CREB3L1 gene (Fig. 6e) for normal and tumor breast samples; the locations of these regions are indicated in Fig. 6f and Additional file 8: Table S4. Some are also shown in more detail in Additional file 3: Figure S2. a Methylation of CREB3L1 DNA in regions 2 and 3 are reduced in breast tumor samples, as compared to normal breast tissue, and this negatively correlates with CREB3L1 mRNA expression (c). Methylation was significantly different between tumor and normal breast tissue in all regions except number 6: region 1 (p = 4.8 × 10^−5^), region 2 (p = 1.2 × 10^−9^), region 3 (p = 4.8 × 10^−20^), region 4 (p = 0.0026), region 5 (p = 2.1 × 10^−6^), region 7 (p = 1.1 × 10^−7^), region 8 (p = 7.6 × 10^−6^), region 9 (p = 1.5 × 10^−18^) and region 10 (p = 3.1 × 10^−11^). b Methylation was significantly different between tumor and normal breast tissue in all regions except number 17: region 11 (p = 1.6 × 10^−7^), region 12 (p = 1.0 × 10^−9^), region 13 (p = 2.3 × 10^−17^), region 14 (p = 0.00058), region 15 (p = 2.6 × 10^−19^), region 16 (p = 1.6 × 10^−45^), region 18 (p = 9.7 × 10^−9^), region 19 (p = 1.8 × 10^−45^) and region 20 (p = 1.1 × 10^−42^). d In addition, methylation in regions 16, 19 and 20 increased substantially in breast tumor samples and positively correlated with CREB3L1 mRNA expression. (PDF 294 kb) [file 13058_2016_672_MOESM9_ESM.pdf]

Fig. S6. Ward *et al.*

**A**

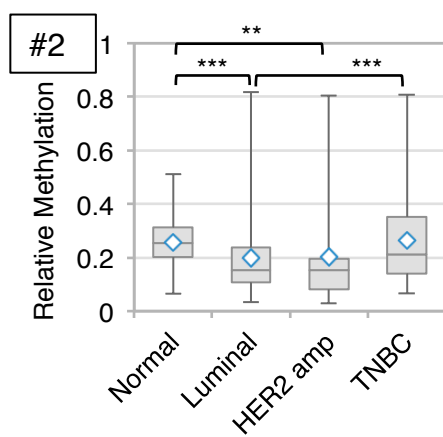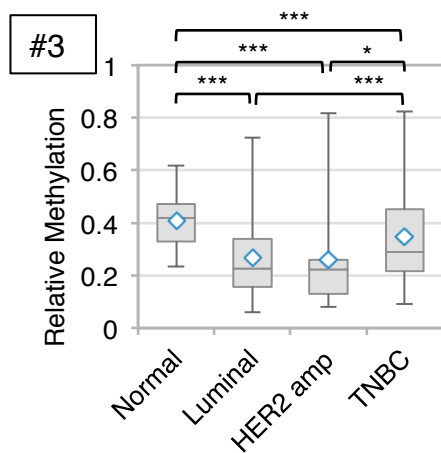

**B**

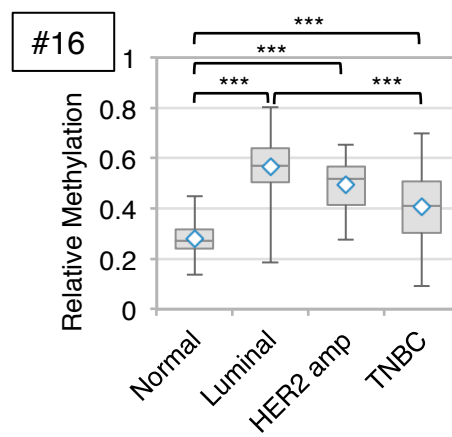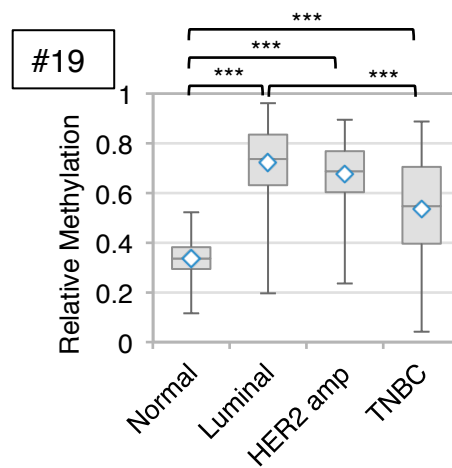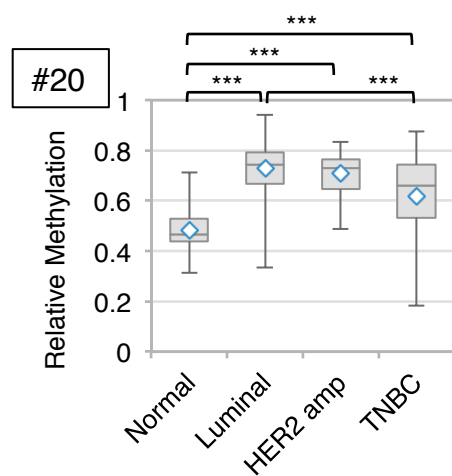

Supplement: Additional file 10: Figure S6. — Methylation within specific CpG regions of the CREB3L1 gene in different breast tumor subtypes. The relative methylation was plotted for each tumor subtype. Methylation in regions 2 and 3 show an inverse correlation with CREB3L1 mRNA expression (found in Fig. 6b), whereas methylation in regions 16, 19 and 20 show a direct correlation with CREB3L1 mRNA expression. For all panels: normal (n = 97), luminal (n = 357), human epidermal growth factor receptor 2 (HER2) amplified (n = 19), triple negative breast cancer (TNBC) (n = 113). Statistical differences were analyzed using post-hoc pairwise comparison: *p <0.05; **p <0.01; ***p <0.001. (PDF 97 kb) [file 13058_2016_672_MOESM10_ESM.pdf]

Fig. S7. Ward *et al.*

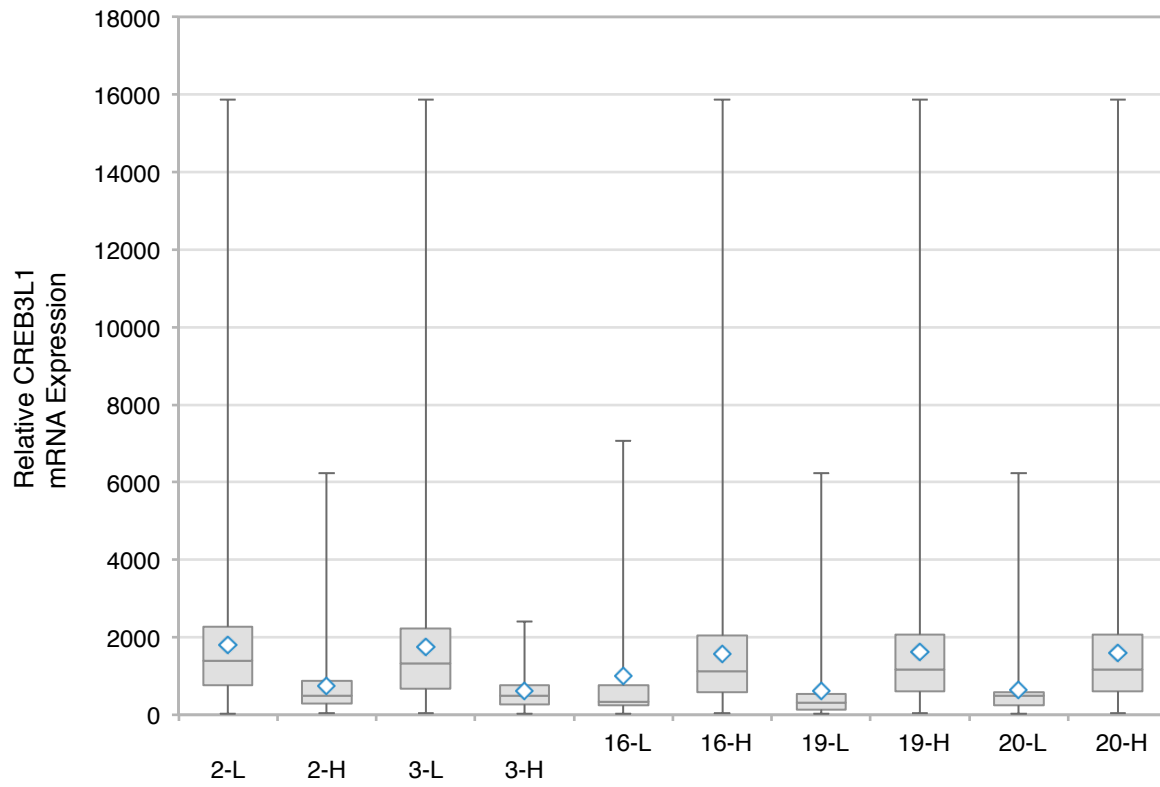

Supplement: Additional file 11: Figure S7. — CREB3L1 mRNA expression varies in breast tumors with CREB3L1 gene methylation in specific regions. For each of the CpG regions shown, tumor samples were divided into low and high methylation groups based on the level of methylation relative to the median methylation in that region in normal breast tissue and plotted according to the level of CREB3L1 mRNA expression for the corresponding samples. Number of samples in each group: site 2: 418-L, 133-H; site 3: 451-L, 100-H; site 16: 114-L, 437-H; site 19: 32-L, 519-H; site 20: 27-L, 524-H. The median methylation in normal breast tissue was as follows: site 2 (0.2556), site 3 (0.4189), site 16 (0.2708), site 19 (0.3352), and site 20 (0.4669). Methylation was significantly different between tumor and normal breast tissue in all regions: region 2 (p = 1.9 × 10^−21^), region 3 (p = 4.0 × 10^−20^), region 16 (p = 4.6 × 10^−5^), region 19 (p = 6.4 × 10^−10^) and region 20 (p = 1.1 × 10^−7^). (PDF 39 kb) [file 13058_2016_672_MOESM11_ESM.pdf]
