# Supplementary material for: Epigenetic silencing of CREB3L1 by DNA methylation is associated with high-grade metastatic breast cancers with poor prognosis and is prevalent in triple negative breast cancers
Source: Breast Cancer Res. 2016 Jan 25;18:12. doi: 10.1186/s13058-016-0672-x (PMC4727399; doi:10.1186/s13058-016-0672-x)
Supplement: Additional file 3: Figure S2. — CREB3L1 promoter region nucleotide sequence with locations of CpG sites. Non-coding nucleotides (lower case) and coding nucleotides (upper case) are shown. CpG sites are highlighted (red and blue). The transcriptional start site (TSS) and the translational start site (coding region) are indicated. The numbering is relative to the translational start site (+1). The CpG island (−118 to +554), as determined using the USCS genome browser, is located within the boxed and shaded region. For the analysis of methylation in breast tumor samples from the The Cancer Genome Atlas database the location of the various cg probes are numbered, labeled and indicated with lines either below or above the sequence. (PDF 48 kb) [file 13058_2016_672_MOESM3_ESM.pdf]

Fig. S2. Ward *et al.*

#14 = cg24825027

ttcgtgcaggctttagtataaggctggaaggatggaaatagctccctcatcaatttacgg -715

#1 = cg12256550

gacagtggttggtgaagccttaaaatgccaatgttccaggaacactgttatcggttccag -655

gactattgttgctttaagcccccaccttgagcattcagagaagagagattactaagacga -595

#4 = cg24644201

agttgcagatgtctctttaaaaaaggagagaggattcctcgagccacgccctcttctcat -535

#6 = cg25626312 #5 = cg20981182

taatattaaattattgtgaatattgatgtggggcgtggcgccttggccagctgggctag -475

TSS (-451)

ccagccaggggttcccggtttcacagagaggaaagtgacagaagacgtgcggagggagac -415

#7 = cg07700369

gcagagacagaggagaggccggcagccacccagtctcggggagcacttagctccccgc -355

cccggctcccaccctgtccggggggctcctgaagccctcagccccaacccccggggctcccc -295

atggaagccagctgtgccccaggaggagcaggaggaggtggagtcggctgaatgcccacg -235

gtgcgcccgggcccctgagcccatcccgctcctagccgctgccctaaggccccgcgcg -175

ccccgcgccccccacccgggcgcgcccgcctccgtccgcccctccccgggggcttcgcc -115

#8 = cg03044069

ccggacctgcccccgcccgtttgccagcgctcaggcaggagctctggactgggcgcgcc -55

5`UTR Start site (coding)

gccgccctggagtgagggaagcccagtggaaggggtcccgggagccggctgcgATGGAC 6

-1

GCCGTCTTGGAACCCTTCCCGGCCGACAGGCTGTTCCCCGGATCCAGCTTCCTGGACTTG 66

exon 1 intron 1

GGGGATCTGAACGAGTCGGACTTCCTCAACAATGCGGTAAGATGAAGGGTCTCCGTTCCC 126

GTTCCACCCCTCGGCACCCGTCCTCGCGGCGCGCCTGGGCCCCTAGAAGGACCCGACTAC 186

ACATCACTGGGCAGGAGCCGGGGAGAGGGTTCAGCGCAGGGTCTCCAGGAGCGACATGTG 246

#2 = cg15917625

TTTGGAGCTAAGCGCCCCTCCTGGGGGCTCAATCTTTGAATACACTGGCCTCCACCTTCT 306

AGGGGGAAGGGGCCATCGAGGTATCGGGTCCGTCCGATCCTCGGATCTGAGCTCAAGAGA 366

CACCAATCCCGCCCTCCACCCACGTCTTCTAGCCGTCCCCAACCCACCCCAGGTTCCAGG 426

ACCAGACTGGGCGCAAAGTGGCAGCGCCCTTTCCTGCGCCTCCCTTGGGGCTTCGGTGGC 486

TCATAGGCTGGATCTCCGCTGGGGGGCGCACCGGGGAACGTTCAGAGGGCCTGAGACCCG 546

#3 = cg06859463

ACCCCTCGGGGAAGCCAGGCCCAGAAATTTCAGGGTGCTACCCTGGCAGCCCCAGGGAAT 606
