# Supplementary material for: Epigenetic silencing of CREB3L1 by DNA methylation is associated with high-grade metastatic breast cancers with poor prognosis and is prevalent in triple negative breast cancers
Source: Breast Cancer Res. 2016 Jan 25;18:12. doi: 10.1186/s13058-016-0672-x (PMC4727399; doi:10.1186/s13058-016-0672-x)
Supplement: Additional file 5: Table S2. — Site-specific methylation data for a panel of 40 breast cancer and 4 non-tumorigenic breast cell lines. (PDF 58 kb) [file 13058_2016_672_MOESM5_ESM.pdf]

Table S2. Site-specific methylation data for panel of 40 breast cancer and normal breast cell lines.

CpG Site -> Cell Lines:	Subtype:	-429	-425	-415	-395	-378	-357	-352	-336	-304	-250	-236	-231	-227	-207	-197	-180	-178	-176	-171	-169	-158	-152	-150	-147	-141	-137	-126	-118	-113	-100	-96	-86	-60	-58	-55	-52	-15	-8	-2	6	9	26	30	45	78	83	101	120	126	138	145	151	153	156	158	180	205	221	238	259	#	score	%
MCF10F	normal	1	1	2	1	1	0	0	0	0	1	1	1	1	1	1	1	2	1	1	1	0	1	1	1	1	1	0	0	0	0	0	0	2	2	2	2	0	0	0	0	0	0	1	2	0	2	2	2	2	2	2	2	2	2	2	2	2	2	2	2	42	32.0	53
MCF12A	normal	2	1	2	1	1	1	0	0	2	1	2	2	2	2	2	1	1	1	2	0	0	0	0	0	0	0	0	0	0	0	0	1	2	2	2	2	1	1	1	1	1	1	1	1	1	2	2	2	2	2	2	2	2	2	2	2	2	2	2	2	46	37.0	62
184B5	normal	2	2	2	0	2	2	2	0	2	2	2	2	2	2	2	2	2	2	2	1	0	1	1	1	1	1	0	2	0	2	2	2	2	2	2	2	0	0	1	1	1	0	0	1	0	0	1	2	2	2	2	2	2	2	2	2	2	2	2	2	49	43.5	73
MCF10A	normal	2	2	2	2	1	2	1	1	1	1	2	2	2	2	2	2	2	2	2	2	2	2	2	2	2	2	1	2	0	2	0	2	2	2	2	2	2	2	2	2	2	1	1	1	2	2	2	2	2	2	2	2	2	2	2	2	2	2	2	2	58	53.5	89
CAMA1	Luminal	0	0	0	0	0	0	0	0	0	0	0	0	0	0	0	0	0	0	0	0	0	0	0	0	0	0	0	0	0	0	0	0	0	0	0	0	0	0	0	0	0	0	0	0	0	0	0	0	0	0	0	0	0	0	0	0	0	0	0	0	0	0.0	0
HCC1500	Luminal	0	0	0	0	0	0	0	0	0	0	0	0	0	0	0	0	0	0	0	0	0	0	0	0	0	0	0	0	0	0	0	0	0	0	0	0	0	0	0	0	0	0	0	0	0	0	0	0	0	0	0	0	0	0	0	0	0	0	0	0	0	0.0	0
ZR-75-1	Luminal	0	0	0	0	0	0	0	0	0	0	0	0	0	0	0	0	0	0	0	0	0	0	0	0	0	0	0	0	0	0	0	0	0	0	0	0	0	0	0	0	0	0	0	0	0	0	0	0	0	0	0	0	0	0	0	0	0	0	0	0	0	0.0	0
BT483	Luminal	0	0	0	0	0	0	0	0	0	0	0	0	0	0	0	0	0	0	0	0	0	0	0	0	0	0	0	0	0	0	0	0	1	1	1	2	0	0	0	0	0	0	0	0	0	0	0	1	1	1	0	0	1	2	2	1	1	0	2	2	14	9.5	16
MDA-MB-134	Luminal	0	0	0	0	0	0	0	0	0	0	0	0	0	0	0	0	0	0	0	0	0	0	0	0	0	0	0	0	0	0	0	0	0	0	0	0	1	2	2	1	1	1	1	1	2	2	2	2	2	2	2	2	2	2	2	2	2	2	2	2	24	21.0	35
MDA-MB-415	Luminal	0	0	0	0	0	0	0	0	0	1	1	1	2	0	0	0	0	0	0	0	0	0	0	0	0	0	0	0	0	0	0	0	0	0	0	0	1	1	1	1	1	1	1	1	0	1	2	2	2	2	2	2	2	2	2	2	2	2	2	2	27	21.0	35
HCC1428	Luminal	0	0	0	0	0	0	0	0	0	0	0	0	0	0	0	0	0	0	0	0	0	0	0	0	0	0	0	0	0	1	0	1	2	2	2	2	1	1	1	1	1	0	0	0	0	1	1	2	2	2	2	2	2	2	2	2	2	2	2	2	26	21.5	36
MDA-MB-175	Luminal	0	0	0	0	0	0	0	0	0	0	0	0	0	0	0	0	0	0	0	0	0	0	0	0	0	0	0	0	0	1	0	0	1	1	1	1	1	1	1	1	1	1	1	1	1	2	2	2	2	2	2	2	2	2	2	2	2	2	2	2	31	22.0	37
T47D	Luminal	0	0	0	0	0	0	0	0	0	0	0	0	0	0	0	0	1	2	1	0	0	0	0	0	0	0	0	0	0	0	0	0	1	1	1	1	1	1	1	1	1	1	1	1	1	1	2	2	2	2	2	2	2	2	2	2	2	2	2	2	31	23.0	38
HCC2157	Luminal	0	0	0	0	0	0	0	0	0	0	0	0	0	1	2	2	2	2	0	0	0	0	0	0	0	0	0	0	0	1	1	1	2	2	2	2	0	0	1	0	0	0	0	0	0	1	2	2	2	2	2	2	2	2	2	2	2	2	2	2	28	25.0	42
MCF7	Luminal	2	2	2	2	2	1	2	1	0	2	2	2	2	2	2	2	2	2	2	1	0	1	1	1	2	1	0	2	1	2	2	2	2	2	2	2	2	2	2	2	2	2	2	1	2	2	2	2	2	2	2	2	2	2	2	2	2	2	2	2	57	52.5	88
BT474	HER2+	0	0	0	0	0	0	0	0	0	0	0	0	0	0	0	0	0	0	0	0	0	0	0	0	0	0	0	0	0	0	0	0	0	0	0	0	0	0	0	0	0	0	0	0	0	0	0	0	0	0	0	0	0	0	0	0	0	0	0	0	0	0.0	0
HCC202	HER2+	0	0	0	0	0	0	0	0	0	0	0	0	0	0	0	0	0	0	0	0	0	0	0	0	0	0	0	0	0	0	0	0	0	0	0	0	0	0	0	0	0	0	0	0	0	0	0	0	0	0	0	0	0	0	0	0	0	0	0	0	0	0.0	0
HCC1419	HER2+	0	0	0	0	0	0	0	0	0	0	0	0	0	0	0	0	0	0	0	0	0	0	0	0	0	0	0	0	0	0	0	0	0	0	0	0	0	0	0	0	0	0	0	0	0	0	0	0	0	0	0	0	0	0	0	0	0	0	0	0	0	0.0	0
UACC-812	HER2+	0	0	0	0	0	0	0	0	0	0	0	0	0	0	0	0	0	0	0	0	0	0	0	0	0	0	0	0	0	0	0	0	0	0	0	0	0	0	0	0	0	0	0	0	0	0	0	0	0	0	0	0	0	0	0	0	0	0	0	0	0	0.0	0
UACC-893	HER2+	0	0	0	0	0	0	0	0	0	0	0	0	0	0	0	0	0	0	0	0	0	0	0	0	0	0	0	0	0	0	0	0	0	0	0	0	0	0	0	0	0	0	0	0	0	0	0	0	0	0	0	0	0	0	0	0	0	0	0	0	0	0.0	0
ZR-75-30	HER2+	0	0	0	0	0	0	0	0	0	0	0	0	0	0	0	0	0	0	0	0	0	0	0	0	0	0	0	0	0	0	0	0	0	0	0	0	0	0	0	0	0	0	0	0	0	0	0	0	0	0	0	0	0	0	0	0	0	0	0	0	0	0.0	0
HCC1954	HER2+	0	0	0	0	0	0	0	0	0	0	0	0	0	0	0	0	0	0	0	0	0	0	0	0	0	0	0	0	0	0	0	0	0	0	0	0	0	0	0	0	0	0	0	0	0	0	0	1	1	1	1	1	1	1	1	1	1	1	1	2	13	7.0	12
SK-BR-3	HER2+	0	0	0	0	0	0	0	0	0	0	0	0	0	0	0	0	0	0	0	0	0	0	0	0	0	0	0	0	0	0	0	0	2	2	2	2	1	1	1	1	1	1	1	0	0	1	0	2	2	2	2	2	2	2	2	2	2	2	2	2	25	21.0	35
MDA-MB-361	HER2+	0	0	0	0	0	0	0	0	0	1	0	1	0	0	0	0	0	0	1	1	0	1	1	1	0	0	0	0	0	2	1	1	2	2	2	2	0	0	0	0	0	0	0	0	0	0	1	2	2	2	2	2	2	2	2	2	2	2	2	2	32	23.0	38
AU565	HER2+	0	0	0	0	0	0	0	0	0	0	0	0	0	2	1	1	1	1	0	0	0	0	0	0	0	0	0	0	0	0	0	1	2	2	2	2	1	1	1	1	1	0	0	0	0	0	2	2	2	2	2	2	2	2	2	2	2	2	1	2	29	23.5	39
HCC2218	HER2+	0	0	0	0	0	0	0	0	0	0	0	0	0	0	0	0	0	0	0	0	0	0	0	0	0	0	0	0	0	0	0	0	1	2	2	2	1	1	1	1	2	1	1	2	2	2	2	2	2	2	2	2	2	2	2	2	2	2	2	2	28	24.5	41
HCC1569	HER2+	1	2	2	2	1	1	1	2	1	1	2	2	2	2	2	2	2	2	1	1	1	1	2	2	2	2	1	2	1	2	1	2	2	2	2	2	2	2	2	2	2	2	2	1	1	2	2	2	2	2	2	2	2	2	2	2	2	2	2	2	60	52.5	88
BT549	TN	0	0	0	0	0	0	0	0	0	0	0	0	0	0	0	0	0	0	0	0	0	0	0	0	0	0	0	0	0	0	0	0	0	0	0	0	0	0	0	0	0	0	0	0	0	0	0	0	0	0	0	0	0	0	0	0	0	0	0	0	0	0.0	0
Hs578T	TN	0	0	0	0	0	0	0	0	0	0	0	0	0	0	0	0	0	0	0	0	0	0	0	0	0	0	0	0	0	0	0	0	0	0	0	0	0	0	0	0	0	0	0	0	0	0	0	0	0	0	0	0	0	0	0	0	0	0	1	1	2	1.0	2
MDA-MB-231	TN	0	0	0	0	0	0	0	0	0	0	0	0	0	0	1	1	1	1	0	0	1	1	1	1	0	0	0	0	1	1	1	0	0	0	1	1	0	0	0	0	0	0	0	0	0	0	0	1	1	1	1	2	2	2	2	2	2	2	2	2	26	17.5	29
HCC70	TN	0	0	0	0	0	0	0	0	0	0	0	0	0	2	2	0	0	0	0	0	0	0	0	0	0	0	0	0	0	2	1	2	2	2	1	1	0	0	0	0	0	0	0	0	0	0	2	2	1	1	1	1	2	2	2	2	1	2	2	2	23	19.0	32
DU4475	TN	2	2	2	1	1	0	0	0	0	0	1	2	1	1	1	1	1	1	1	0	0	0	0	0	0	0	0	0	0	1	1	2	2	2	2	1	0	0	1	0	1	0	1	0	0	0	0	1	1	0	0	0	0	0	0	0	0	2	1	2	29	19.5	33
HCC38	TN	0	0	0	0	0	0	0	0	0	0	0	0	0	1	1	0	0	0	0	0	0	0	0	0	0	0	0	0	0	2	0	1	2	2	2	2	0	0	0	0	0	0	0	0	0	0	1	2	2	2	2	2	2	2	2	1	2	2	2	2	22	19.5	33
HCC1187	TN	0	0	0	0	0	0	0	0	0	0	0	0	0	0	0	0	0	0	0	0	0	0	0	0	0	0	0	1	0	1	1	1	2	2	2	2	0	0	0	0	1	0	0	0	0	1	1	2	2	2	2	2	2	2	2	2	2	2	2	2	24	20.5	34
MDA-MB-436	TN	0	0	0	0	0	0	0	0	0	0	0	0	0	0	0	0	0	0	0	0	0	1	1	0	0	0	0	0	0	0	0	0	0	0	0	0	1	1	1	1	1	1	1	1	1	2	2	2	2	2	2	2	2	2	2	2	2	2	2	2	26	20.5	34
MDA-MB-453	TN	0	0	0	0	0	0	0	0	0	0	0	0	0	0	0	0	0	0	0	0	0	0	0	0	0	0	0	0	0	0	0	0	0	0	1	1	1	1	1	1	1	1	1	1	2	2	2	2	2	2	2	2	2	2	2	2	2	2	2	2	26	21.0	35
HCC1937	TN	0	0	0	0	0	0	0	0	0	0	0	0	0	0	0	0	0	0	0	0	1	1	1	1	1	1	1	1	1	1	1	2	2	2	2	2	0	0	1	0	1	1	0	0	0	1	1	2	2	2	2	1	2	2	2	1	2	2	2	2	34	25.0	42
MDA-kb2	TN	0	0	0	0	0	0	0	0	0	0	0	0	2	0	0	0	0	0	0	0	0	0	0	0	0	0	0	0	0	0	1	1	0	2	1	2	1	1	2	2	2	1	1	2	2	2	2	2	2	2	2	2	2	2	2	2	2	2	2	2	30	27.5	46
HCC1806	TN	1	2	1	1	1	1	1	1	1	1	1	1	0	1	2	1	2	2	1	2	0	1	1	1	1	1	1	1	1	2	1	2	2	2	2	2	1	2	2	2	2	0	0	0	0	2	2	2	2	2	2	2	2	2	2	2	2	2	2	2	54	42.0	70
BT20	TN	1	1	1	0	1	0	0	0	0	1	2	2	2	2	2	2	2	2	2	2	2	2	2	2	2	2	2	2	2	2	2	2	2	2	2	2	2	2	2	2	2	2	2	2	2	2	2	2	2	2	2	2	2	2	2	2	2	2	2	2	45	42.5	71
HCC1395	TN	2	2	2	2	2	1	0	0	0	0	1	2	1	2	2	2	2	2	2	2	1	0	0	2	1	0	1	1	2	1	2	2	2	2	2	2	1	2	2	2	2	0	2	0	1	2	2	2	2	2	2	2	2	2	2	2	2	2	2	2	51	46.0	77
MDA-MB-157	TN	2	2	2	2	2	2	1	1	1	2	2	2	2	2	2	2	1	2	1	1	0	1	1	1	1	1	0	1	1	1	1	1	1	1	1	1	2	2	2	2	2	2	2	2	2	2	2	2	2	2	2	2	2	2	2	2	2	2	2	2	58	48.0	80
HCC1599	TN	2	2	2	2	2	2	1	1	1	2	1	2	2	2	2	2	2	2	2	2	1	1	2	1	1	1	2	2	1	2	2	2	2	2	2	2	2	2	2	2	2	2	2	2	2	2	2	2	2	2	2	2	2	2	2	2	2	2	2	2	60	55.0	92
MDA-MB-468	TN	2	2	2	2	2	2	2	1	2	2	2	2	2	2	2	2	2	2	2	2	0	1	2	2	2	2	0	2	1	2	2	2	2	2	2	2	2	2	2	2	2	2	2	2	2	2	2	2	2	2	2	2	2	2	2	2	2	2	2	2	58	56.5	94
**# methylated**		13	13	13	11	13	10	8	7	8	13	14	15	14	17	18	16	17	17	15	12	7	14	14	14	12	11	7	12	10	20	17	20	27	27	29	29	21	21	25	22	25	18	20	18	16	24	29	33	33	32	31	31	32	32	32	32	32	32	34	34			
**Frequency (%)**		30	30	30	25	30	23	18	16	18	30	32	34	32	39	41	36	39	39	34	27	16	32	32	32	27	25	16	27	23	45	39	45	61	61	66	66	48	48	57	50	57	41	45	41	36	55	66	75	75	73	70	70	73	73	73	73	73	73	77	77			

= not determined

0 = not methylated

1 = somewhat methylated

2 = predominantly methylated

# - the number of distinct methylation sites, either somewhat or predominantly methylated, over the entire region analysed for that cell line.

Score - the weighted cummulative methylation that only includes data from sites -429 to +259. Sites scored as somewhat (1) and predominantly (2) methylated are given a value of 0.5 and 1 respectively.

% methylation - an estimate of level of methylation observed that only includes data from sites -429 to +259, analyzed for all cell lines. % methylation = Score/(60 CpG sites total) *100.

Frequency (%) - the percent of cell lines showing methylation at that specific site; Frequency (%) = # methylated/44*100
