# Supplementary material for: Epigenetic silencing of CREB3L1 by DNA methylation is associated with high-grade metastatic breast cancers with poor prognosis and is prevalent in triple negative breast cancers
Source: Breast Cancer Res. 2016 Jan 25;18:12. doi: 10.1186/s13058-016-0672-x (PMC4727399; doi:10.1186/s13058-016-0672-x)
Supplement: Additional file 7: Table S3. — Site-specific methylation data for the 35 breast tumor samples that contained methylated sites. (PDF 46 kb) [file 13058_2016_672_MOESM7_ESM.pdf]

Table S3. Site-specific methylation data for 35 human breast tumor samples.

CpG Site -> Patient:	-429	-425	-415	-395	-378	-357	-352	-336	-304	-250	-236	-231	-227	-207	-197	-180	-178	-176	-171	-169	-158	-152	-150	-147	-141	-137	-126	-118	-113	-100	-96	-86	-60	-58	-55	-52	-15	-8	-2	6	9	26	30	45	78	83	101	120	126	138	145	151	153	156	158	180	205	221	238	259	#	score	%
15618																											0	0	0	0	0	0	0	0	1	0	0	0	0	0	0	0	0	0	0	0	0	0	0	0	0	0	0	0	0	0	0	0	0		1	0.0	0
15245																																					0	0	0	0	0	0	0	0	0	0	0	0	0	0	0	0	0	0	0	0	1	2	0		2	1.5	7
16834	0	0	0	0	0	0	0	0	0	0	0	0	0	0	0	0	0	0	0	0	0	0	0	0	0	0	0	0	0	0	0	0	0	0	0	0	0	0	0	0	0	0	0	0	0	0	0	0	0	0	0	0	0	0	0	0	1	1	2	1	4	2.0	9
16731	0	0	0	0	0	0	0	0	0	0	0	0	0	1	0	0	0	0	0	0	0	0	0	0	0	0	0	0	0	0	0	0	0	0	0	0	0	0	0	0	0	0	0	0	0	0	0	0	0	0	0	0	0	0	0	0	1	2	1	1	5	2.0	9
16748	0	0	0	0	0	0	0	0	0	0	0	0	0	0	0	0	0	0	0	0	0	0	0	0	0	0	0	0	0	0	0	0	0	0	0	0	0	0	0	0	0	0	0	0	0	0	0	0	0	0	0	0	0	0	0	0	1	2	1	2	4	2.0	9
13307	0	0	0	0	0	0	0	0	0	0	0	0	0	0	0	0	0	0	0	0	0	0	0	0	0	0	0	0	0	0	0	0	0	0	0	0	0	0	0	0	0	0	0	0	0	0	0	0	0	0	0	0	0	0	0	0	1	2	2	0	3	2.5	11
11913	0	0	0	0	0	0	0	0	0	0	0	0	0	0	0	0	0	0	0	0	0	0	0	0	0	0	0	0	0	0	0	0	0	0	0	0	0	0	0	0	0	0	0	0	0	0	0	1	0	0	0	0	0	2	1	0	0	1	1	2	6	3.0	13
13413	0	0	0	0	0	0	0	0	0	0	0	0	0	0	0	0	0	0	0	0	0	0	0	0	0	0	0	0	0	0	0	0	1	1	1	1	0	0	0	0	0	0	0	0	0	0	0	0	0	0	0	1	1	1	1	1	1	1	1	2	13	4.0	17
14991	0	0	0	0	0	0	0	0	0	0	0	0	0	0	0	0	0	0	0	0	0	0	0	0	0	0	0	0	0	0	0	0	0	0	0	0	0	0	0	0	0	0	0	0	0	0	0	1	1	0	0	1	1	1	1	0	1	0	1	2	9	5.0	22
16525	0	0	0	0	0	0	0	0	0	0	0	0	0	0	0	0	0	0	0	0	0	0	0	0	0	0	0	0	0	0	0	0	1	1	1	1	0	0	1	0	1	0	0	0	0	0	0	0	0	0	0	1	1	1	1	1	1	2	2	2	15	6.0	26
15830						0	0	0	0																										0	0	0	0	0	0	0	0	0	0	0	0	0	1	1	0	0	1	1	1	2	1	1	2	2		10	6.5	28
8081	0	0	0	0	0	0	0	0	0	0																											0	0	0	0	0	0	0	0	0	0	0	1	0	1	2	1	2	1	1	1	0	2	2		10	6.5	28
16610	0	0	0	0	0	0	0	0	0	0	0	0	0	0	0	0	0	0	0	0	0	0	0	0	0	0	0	0	0	0	0	0	0	0	0	0	0	0	0	0	0	0	0	0	0	0	0	1	1	1	0	1	1	2	2	1	1	1	1	2	11	6.5	28
12281																																					0	0	1	0	0	0	0	0	0	0	0	1	1	1	1	1	1	1	1	1	1	2	2		13	7.5	33
14642											0	0	0	0	0	0	0	0	0	0	0	0	0	0	0	0	0	0	0	0	0	0	0	0	0	0	0	0	0	0	0	0	0	0	0	0	0	1	1	1	2	1	2	2	2	0	1	1	1		11	7.5	33
16557	0	0	0	0	0	0	0	0	0	0	0	0	0	0	0	0	0	0	0	0	0	0	0	0	0	0	0	0	0	0	0	0					0	0	0	0	0	0	0	0	0	0	0	0	1	1	1	1	1	1	2	1	2	2	2	2	12	7.5	33
13869																																					0	0	0	0	0	0	0	0	0	0	0	1	1	1	1	1	2	2	2	1	1	2	2		12	8.5	37
16640	0	0	0	0	0	0	0	0	0	0	0	0	0	0	0	0	0	0	0	0	0	0	0	0	0	0	0	0	0	0	0	0	0	0	0	0	0	0	0	0	0	0	0	0	0	0	0	1	1	1	1	1	1	2	2	1	2	2	2	2	13	8.5	37
16641	0	0	0	0	0	0	0	0	0	0	0	0	0	0	0	0	0	0	0	0	0	0	0	0	0	0	0	0	0	0	0	0	0	0	0	0	0	0	0	0	0	0	0	0	0	0	0	1	1	1	1	1	1	2	2	1	2	2	2	2	13	8.5	37
14124	0	0	0	0	0	0	0	0	0	0	0	0	0	0	0	0	0	0	0	0	0	0	0	0	0	0	0	0	0	0	0	0	1	1	1	1	0	0	0	0	0	0	0	0	0	0	0	1	1	1	1	1	1	1	2	1	2	2	2	2	17	10.0	43
15876	0	0	0	0	0	0	0	0	0	0	0	0	0	0	0	0	0	0	0	0	0	0	0	0	0	0	0	0	0	0	0	0	0	0	0	0	0	0	0	0	0	0	0	0	0	0	0	1	1	2	1	2	2	2	2	1	2	2	2	2	13	10.0	43
15237						0	0	0	0												0	0	0	0	0	0	0	0	0	0	0	0	0	0	0	0	0	0	0	1	1	0	0	0	0	0	1	2	2	2	1	1	1	2	2	0	1	2	2		14	10.5	46
14697																																					0	0	1	0	1	0	0	0	0	1	0	1	1	1	1	2	2	2	2	1	2	2	2		15	11.0	48
11903	0	0	0	0	0	0	0	0	0	0	0	0	0	0	0	0	0	0	0	0	0	0	0	0	0	0	0	0	0	0	0	0	0	0	0	0	0	0	0	0	0	0	0	0	0	0	0	2	1	2	2	1	2	2	2	2	2	2	2	2	13	11.0	48
16651	0	0	0	0	0	0	0	0	0	0	0	0	0	0	0	0	0	0	0	0	0	0	0	0	0	0	0	0	0	0	0	0	0	0	0	0	0	0	0	0	0	0	0	0	0	0	0	2	2	2	1	2	2	2	2	1	2	2	2	2	13	11.0	48
12159																																					0	0	1	0	0	0	0	0	0	0	2	2	2	2	1	1	2	2	2	1	1	2	2		14	11.5	50
16558	0	0	0	0	0	0	0	0	0	0	0	0	0	0	0	0	0	0	0	0	0	0	0	0	0	0	0	0	0	0	0	0	0	0	0	0	0	0	0	0	0	0	0	0	0	0	1	2	2	2	2	2	2	2	2	2	2	2	2	2	14	12.5	54
13123	0	0	0	0	0	0	0	0	0	0	0	0	0	0	0	0	0	0	0	0	0	0	0	0	0	0	0	0	0	0	0	0	1	1	1	1	0	0	0	0	0	0	0	0	0	0	2	2	2	2	2	2	2	2	2	1	2	2	2	2	18	12.5	54
16331				0	0	0	0	0	0																				0	1	1	1	1	1	1	1	0	0	1	1	1	0	0	0	0	1	1	2	2	2	2	1	2	2	2	1	2	2	2	2	25	13.5	59
16377			0	0	0	0	0	0	0	0	0	0	1																								0	0	0	0	0	0	0	0	0	0	1	1	1	1	1	1	2	2	2	2	2	2	2		14	14.0	61
16405	0	0	0	0	0	0	0	0	0	0	0	0	0	0	0	0	0	0	0	0	0	0	0	0	0	0	0	0	0	1	1	0	1	1	1	1	0	0	1	0	1	0	0	0	0	1	2	2	2	2	2	2	2	2	2	2	2	2	2	2	23	14.5	63
14877																																					0	0	0	0	1	1	1	1	1	2	2	2	2	2	2	1	2	2	2	2	1	2	2		19	15.5	67
15175																																					0	0	0	1	1	1	1	1	1	1	2	2	2	2	2	2	2	2	2	2	2	2	2		20	16.5	72
16529	0	0	0	0	0	0	0	0	0	0	0	0	0	0	0	0	0	0	0	0	0	0	0	0	0	0	0	0	0	0	0	0	1	1	1	1	1	1	1	1	1	1	1	1	0	1	1	2	2	2	2	2	2	2	2	2	2	2	2	2	28	17.0	74
16667	0	0	0	0	0	0	0	0	0	0	0	0	1	2	2	1	1	1	1	0	0	0	0	0	1	1	0	0	0	1	1	1	2	2	2	2	2	2	2	1	2	2	1	0	0	1	2	2	2	2	2	2	2	2	2	1	2	2	2	2	38	19.0	83
# methylated	0	0	0	0	0	0	0	0	0	0	0	0	2	2	1	1	1	1	1	0	0	0	0	0	1	1	0	0	0	3	3	3	8	8	8	8	2	2	8	5	9	4	4	3	2	7	11	26	25	24	23	28	28	29	29	25	32	33	33	21			
# determined	22	22	23	24	24	24	24	24	26	23	23	23	23	22	22	22	22	22	22	22	23	23	23	23	23	23	24	24	25	25	25	25	24	24	24	24	35	35	35	35	35	35	35	35	35	35	35	35	35	35	35	35	35	35	35	35	35	35	35	22			
Frequency (%)	0	0	0	0	0	0	0	0	0	0	0	0	9	9	5	5	5	5	5	0	0	0	0	0	4	4	0	0	0	12	12	12	33	33	33	33	6	6	23	14	26	11	11	9	6	20	31	74	71	69	66	80	80	83	83	71	91	94	94	95			

(blank) = not determined
0 = not methylated
1 = somewhat methylated
2 = predominantly methylated

# - the number of distinct methylation sites, either somewhat or predominantly methylated, over the entire region analysed for that tumor sample.

Score - the weighted cummulative methylation that only includes data from sites -15 to +238, where all data is available for all samples. Sites scored as somewhat (1) and predominantly (2) methylated are given a value of 0.5 and 1 respectively. Note this excludes site 259, since data was not available for all samples for this site.

% methylation - an estimate of level of methylation observed that only includes data from sites -15 to +238, where all data is available for all samples. % methylation = Score/(23 CpG sites total) *100.

Frequency (%) - the percent of tumors showing methylation at that specific site; Frequency (%) = # methylated/(# determined) x 100
