# Supplementary material for: Epigenetic silencing of CREB3L1 by DNA methylation is associated with high-grade metastatic breast cancers with poor prognosis and is prevalent in triple negative breast cancers
Source: Breast Cancer Res. 2016 Jan 25;18:12. doi: 10.1186/s13058-016-0672-x (PMC4727399; doi:10.1186/s13058-016-0672-x)
Supplement: Additional file 8: Table S4. — Probe information used in The Cancer Genome Atlas (TCGA) database to analyze CREB3L1 gene methylation in human breast tumor samples. (PDF 38 kb) [file 13058_2016_672_MOESM8_ESM.pdf]

Table S4. Probe information used in TCGA to analyze CREB3L1 gene methylation in human breast tumor samples.

#	IlmnID-probe set	Methylated	Location	Feature	Source Sequence	Strand
14	cg24825027	variably	(-772)-(-723)	promoter	CGTGCAGGCTTTAGTATAAGGCTGGAAGGATGGAAATAGCTCCCTCATCA	F
1	cg12256550	variably	(-717)-(-668)	promoter	CGGGACAGTGGTTGGTGAAGCCTTAAAATGCCAATGTTCCAGGAACACTG	F
4	cg24644201	variably	(-597)-(-548)	promoter	GTGGCTCGAGGAATCCTCTCTCCTTTTTTAAAGAGACATCTGCAACTTCG	R
6	cg25626312	variably	(-507)-(-458)	promoter	CGGGAACCCCTGGCTGGCTAGCCCAGCTGGCCAAGGCGCCACGCCCCCAC	R
5	cg20981182	variably	(-495)-(-446)	promoter	TCTCTGTGAAACCGGGAACCCCTGGCTGGCTAGCCCAGCTGGCCAAGGCG	R
7	cg07700369	variably	(-395)-(-346)	5`UTR	GGAGCCGGGGCGGGGGAGCTAAGTGCTCCCCCGAGACTGGGTGGCTGCCG	R
8	cg03044069	variably	(-113)-(-64)	5`UTR	AGTCCAGAGCTCCTGCCTGAGCGCTGGCAAACGGGCGGGGGGCAGGTCCG	R
2	cg15917625	variably	238-287	intron 1	ATTCAAAGATTGAGCCCCCAGGAGGGGCGCTTAGCTCCAAACACATGTCG	R
3	cg06859463	variably	553-602	intron 1	CGGGGAAGCCAGGCCCAGAAATTTCAGGGTGCTACCCTGGCAGCCCCAGG	F
15	cg25963429	variably	1700-1749	intron 1	CGGCCGAGTTCATTCAGAAGAAAGCCCAACTCAGAGCTGCCAAGGGCCGA	R
17	cg16012690	variably	15146-15195	intron 1	AGGGCTGGGATTAGCTGTGTCTGGCACCTCCGAGGGCACAGCAGCCCACG	R
18	cg09709565	variably	16620-16669	intron 1	CGCCCCCTAGGCACAGCAGTGACTCAGACTGAAGGCCCTTCAGCTTGAAA	F
10	cg02364610	variably	17373-17422	intron 1	CGGCCAGGCCATTTCTGCCTCGGCCCCAGACGCAGAAGGGGTCCCATGCA	F
11	cg14547067	variably	17491-17540	intron 1	GCAGCAGCCGGTGGCGGCGTGGAGGGCACCGGAGGAGTGCAGGGCTCGCG	R
13	cg03131732	variably	17812-17861	intron 1	AGACTTTTTCCTATAGTCGTGCCAATATAAGCTCCTTCTCGGCTGTCACG	R
12	cg09896120	variably	17916-17963	intron 1	AGGGTTCTGCAGGGGGTGAGCGTCCTACAGGGGTGCCTTTCCAGGCAACG	R
19	cg20068058	variably	18148-18197	intron 1	GTCTGAGGTCTCCAAAATGGGCTTTCCAGGGATGGGGCCAAGTGGATGCG	F
20	cg22902089	variably	18560-18609	intron 1	CGCCCACCGTCCACCCAGCACCTGAAGAAGCCCGGCGCAAAGAGGACCTG	F
16	cg07008193	variably	18816-18865	intron 1	CGGATCTGGGCTGGACTCCCTGAAGGCCTTGGCTCAGGCAGATGACCCAG	R
9	cg21690489	variably	30549-30598	intron 3	CGCTCCCATCTAGGGATTTTAAGATGCTGCTCATCCTGTGGAATCCCAGG	R
	cg00352031	no	(-1302)-(-1253)	5`UTR	CGGTATACACCCACAGGGCATCATGTGCTCACCTCCACTGTTTCCAGTCT	R
	cg06588284	no	6015-6064	intron 1	TGCCAGGGTAACCCAGATGAAGAAAACACACTTGACCCTCCAGTCAATCG	R
	cg06884875	no	13340-13389	intron 1	CGGACCTGGTTCTAATCCTGGTTCTGCTATACACTGTGCAGGCTTTGGAC	R
	cg16795564	no	3393-3442	intron 1	CGGGTGTGCTGGGCAAACATCCTTGCAGCTTCCTGCTGCTCCCCTCTGAG	R
	cg18857647	no	21524-21573	intron 1	CGCTCACCATTGTTATCTGCAAGGGTGAGCTCAGGATGGGATCAAAAACT	R
	cg04424721	yes; both tumor & normal	42751-42800	intron 10	TTTCTCCTGGAATGGGAAGCTCCAGGAGGCAGGGGCACGAGGCGGATCCG	F

# - number assigned to each region.

IlmnID-probe set - TCGA probe name.

Methylated - if region is methylated in normal and/or tumor breast tissue.

Location - location of the 50 bp probe region within the CREB3L1 gene, relative to the translational start site.

Feature - location of the 50 bp probe region with regards to the gene structure.

Source Sequence - sequence of the 50 bp region analyzed.

Strand - F = forward strand; R = reverse strand, to which the source sequence corresponds.
